# Transient suppression of transplanted spermatogonial stem cell differentiation restores fertility in mice

**DOI:** 10.1016/j.stem.2021.03.016

**Published:** 2021-08-05

**Authors:** Yoshiaki Nakamura, David J. Jörg, Yayoi Kon, Benjamin D. Simons, Shosei Yoshida

**Affiliations:** 1 Division of Germ Cell Biology, National Institute for Basic Biology, National Institutes of Natural Sciences, Higashiyama 5-1, Myodaiji, Okazaki 444-8787, Japan; 2 Laboratory of Animal Breeding and Genetics, Graduate School of Integrated Sciences for Life, Hiroshima University, 1-4-4 Kagamiyama, Higashi-Hiroshima, Hiroshima 739-8528, Japan; 3 Japan Society for the Promotion of Science, 5-3-1 Kojimachi, Chiyoda-ku, Tokyo 102-0083, Japan; 4 Wellcome Trust-Cancer Research UK Gurdon Institute, University of Cambridge, Cambridge CB2 1QN, UK; 5 Cavendish Laboratory, Department of Physics, University of Cambridge, Cambridge CB3 0HE, UK; 6 Department of Applied Mathematics and Theoretical Physics, Centre for Mathematical Sciences, University of Cambridge, Wilberforce Road, Cambridge CB3 0WA, UK; 7 Wellcome Trust-Medical Research Council Stem Cell Institute, University of Cambridge, Cambridge CB2 1QR, UK; 8 Department of Basic Biology, School of Life Science, Graduate University for Advanced Studies (Sokendai), Okazaki 444-8787, Japan

**Keywords:** spermatogenesis, mouse, stem cells, transplantation, repopulation, clonal fate, mathematical model, fertility restoration, retinoic acid, WIN18,446

## Abstract

A remarkable feature of tissue stem cells is their ability to regenerate the structure and function of host tissue following transplantation. However, the dynamics of donor stem cells during regeneration remains largely unknown. Here we conducted quantitative clonal fate studies of transplanted mouse spermatogonial stem cells in host seminiferous tubules. We found that, after a large population of donor spermatogonia settle in host testes, through stochastic fate choice, only a small fraction persist and regenerate over the long term, and the rest are lost through differentiation and cell death. Further, based on these insights, we showed how repopulation efficiency can be increased to a level where the fertility of infertile hosts is restored by transiently suppressing differentiation using a chemical inhibitor of retinoic acid synthesis. These findings unlock a range of potential applications of spermatogonial transplantation, from fertility restoration in individuals with cancer to conservation of biological diversity.

## Highlights

• Following transplantation, spermatogonial stem cells follow stochastic clone dynamics
• Most initially settled stem cells are lost through chance differentiation and death
• Donor cell differentiation can be suppressed using a retinoic acid synthesis inhibitor
• Transient differentiation block boosts repopulation and restores fertility of the host

## Graphical abstract

**Figure:**
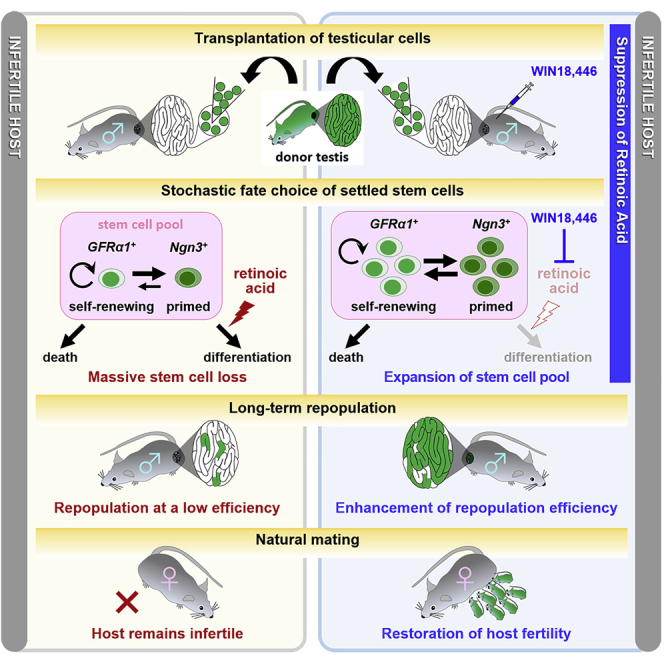

## Teaser

Nakamura et al. analyzed the fate of transplanted mouse spermatogonial stem cells (SSCs) at single-cell resolution. They found that only a small fraction of equipotent SSCs repopulate over the long term and demonstrated a strategy to restore host fertility by transient suppression of donor SSC differentiation using a chemical inhibitor.

## Introduction

Tissue stem cells can restore the impaired structure and function of host tissues following transplantation. In the blood, hematopoietic stem cell transplantation is established as a radical treatment for leukemia, and trials to test the viability of stem cell transplantation in mesenchymal and epithelial tissues are ongoing (De Luca et al., 2019). In germ cells, spermatogonial stem cell (SSC) transplantation has been established in mice (Brinster and Avarbock, 1994; Brinster and Zimmermann, 1994; Figure S1A), promising a wealth of applications such as restoration of fertility of male individuals with cancer after chemotherapy or preservation of genetic diversity (Firlej et al., 2012; Honaramooz and Yang, 2010). However, currently, inefficiency rules out practical applications of this technology.

Beginning with pioneering studies in hematopoiesis, much emphasis has been placed on defining the stem cell potential based on their regenerative capacity after transplantation. In mouse spermatogenesis, stem cell transplantation has been used for quantitative and functional assessment of stem cell potential (Brinster, 2002). However, our knowledge of the fate behavior of individual SSCs and their progenies following transplantation remains poor, limiting the potential to develop new strategies to increase the currently low transplantation efficiencies.

In the transplantation method developed by Brinster, (2002), a single-cell suspension of donor testes is injected into the lumen of the seminiferous tubules of the host animal, whose germ cells have been eliminated by treatment with a cytotoxic reagent (e.g., busulfan) or genetic mutations (e.g., *Kit*^*W/Wv*^). When settled, donor spermatogonia translocate through the tight junction between Sertoli cells onto the basal membrane, where spermatogonia, including SSCs and amplifying progenitors, normally reside (Nagano et al., 1999; Russell, 1990). After 2–3 months, some donor cells are able to repopulate and restore homeostatic sperm production in a segment of host tubules, harboring all stages of spermatogenesis. These local repopulation events manifest as “colonies” that originate from a single founder cell (Kanatsu-Shinohara et al., 2006). In homeostasis, *bona fide* or reconstituted spermatogonia reside in the basal compartment, defined as the gap between the basement membrane and the tight junction between Sertoli cells, whereas meiotic and haploid cells (designated spermatocytes and spermatids, respectively) are located adluminally, forming the stratified organization of tissue (Russell, 1990).

**Figure 1:**
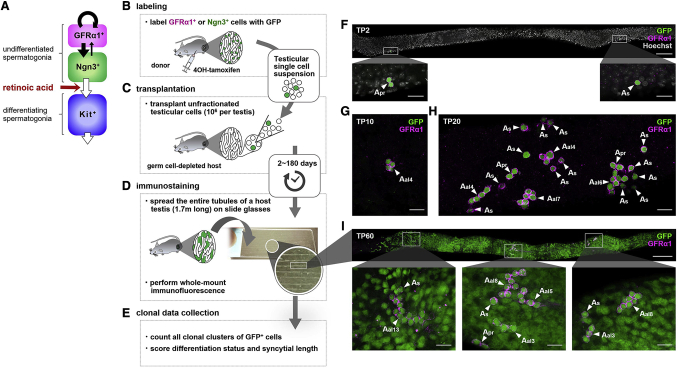
Pulse-transplantation experiments (A) A summary of the hierarchical relationship between spermatogonial subsets. (B–E) Outline of the “pulse-transplantation” experiments. (B and C) GFRα1^+^ and Ngn3^+^ A_undiff_ were pulse labeled with GFP (green) by 4OH-tamoxifen injection (B), and the testes of host animals were dissociated 2 days later and transplanted into germ cell-depleted host testes (C). (D) On TP2–TP180, the entire ∼1.7-m-long tubules of each host testis were collected, aligned on slide glasses, and processed for whole-mount IF, with a part magnified to show tubule alignment. (E) All clonal clusters of GFP^+^ cells in host tubules were scored for the differentiation status of constituent A_undiff_ and, at later time points, for the spatial extent within the seminiferous tubules. (F–I) Examples of the IF images of a part of host tubules at different time points, stained for GFP (green) and GFRα1 (magenta). (F) On TP2, GFP^+^ donor spermatogonia were distributed sparsely (arrowheads in magnified images); they were stained for DNA with Hoechst 33342 (gray). (G and H) Images of surviving clones on TP10 and TP20, respectively. (I) A part of a seminiferous tubule at TP60, showing an entire colony containing GFRα1^+^ cells. Arrowheads indicate GFP^+^/GFRα1^+^ cells with the indicated topologies. Scale bars, 200 μm (F and I, top) and 25 μm (G, H, and magnified images in F and I). Examples of GFRα1- and Ngn3-induced donor clones are shown in (F)–(H) and (I), respectively.

In mice, SSC potential is largely restricted to a small population of undifferentiated spermatogonia (A_undiff_) (de Rooij and Russell, 2000; Ohbo et al., 2003; Russell, 1990; Shinohara et al., 2000). A_undiff_ show heterogeneous composition in their morphology (comprising singly isolated cells [A_s_] and syncytia of two [A_pr_] or more [A_al_] cells connected via intercellular bridges), gene expression, and *in vivo* behavior. During homeostasis, the glial cell line derived neurotrophic factor family receptor alpha 1 (GFRα1)^+^ fraction of A_undiff_ comprises the majority of the self-renewing pool while giving rise to the differentiation-primed, neurogenin3 (Ngn3)^+^ (largely GFRα1^–^/retinoic acid receptor γ [RARγ]^+^/Miwi2^+^) fraction of A_undiff_ (Carrieri et al., 2017; Hara et al., 2014; Ikami et al., 2015). Ngn3^+^ cells rarely self-renew but, instead, differentiate into Kit^+^ differentiating spermatogonia in a manner dependent on retinoic acid (RA) signaling (Figure 1A; Gely-Pernot et al., 2012; Ikami et al., 2015; Nakagawa et al., 2010; van Pelt and de Rooij, 1990). Nevertheless, the Ngn3^+^ (Miwi2 [Piwil4]^+^) fraction of A_undiff_ has the potential to transit reversibly to a GFRα1^+^ state, playing a vital role in recovery following tissue insult (Carrieri et al., 2017; Nakagawa et al., 2007). Following transplantation, repopulation potential is detected widely across the entire A_undiff_ population, albeit with variable efficiency (Carrieri et al., 2017; Garbuzov et al., 2018; Helsel et al., 2017; La et al., 2018; Nakagawa et al., 2007). In contrast, Kit^+^ spermatogonia are committed to differentiation to meiotic spermatocytes and devoid of SSC potential (Ohbo et al., 2003). Most quantitative studies of transplantable SSCs rely on scoring the number of resultant colonies established over the long term (typically 2–3 months), based on the prevailing view that a few potent and definitive SSCs are pre-determined to repopulate with high probability. However, to date, little is known about the dynamics and fate behavior of donor spermatogonia during repopulation.

In homeostasis, GFRα1^+^ cells migrate actively over the basement membrane while undergoing incomplete cell division to form syncytia or double their syncytial length and syncytial fragmentation to give rise to isolated cells or shorter syncytia (Hara et al., 2014). Accordingly, GFRα1^+^ cells maintain their numbers through interconversion between A_s_, A_pr_, and A_al_ states while giving rise to GFRα1^–^ cells, resulting in highly variable fates, as shown by individual GFRα1^+^ cell clones (Hara et al., 2014). However, despite this apparent complexity, the statistics of clonal evolution during homeostatic spermatogenesis can be predicted quantitatively by a minimal model in which GFRα1^+^ cells undergo rounds of cell division, syncytial fragmentation, and differentiation stochastically with defined probabilities (Hara et al., 2014; Klein et al., 2010). As a consequence, in homeostasis, SSC clones are not individually long lived, as would occur if SSCs renewed through invariant asymmetric division. Instead, individual SSC clones follow highly divergent fates where some expand and persist, contributing to long-term self-renewal, whereas others are lost through chance differentiation (Hara et al., 2014). This raises the question of whether, following transplantation in the emptied niche microenvironment, donor spermatogonia exhibit similarly stochastic and divergent fates or whether stereotypic fate behavior enables a definitive subpopulation to repopulate with high probability.

Here, to define the dynamics of donor SSCs after transplantation, we quantified the clonal fate of transplanted GFRα1^+^ and Ngn3^+^ spermatogonia over the long term (up to 180 days). Based on a quantitative analysis of the clone dynamics, we further developed and applied a strategy to significantly enhance the repopulation efficiency by tuning the post-transplantation fate of donor SSCs.

## Results

### Post-transplantation fate analysis of GFRα1^+^ and Ngn3^+^ spermatogonia

To resolve the identity of SSCs that restore spermatogenesis following transplantation and to define their dynamics during repopulation, we performed quantitative clonal fate analysis of donor A_undiff_ in host testes of adult mice (Figures 1B–1I). GFRα1^+^ or Ngn3^+^ cells were pulse labeled by administering 4-hydroxy (4OH-)tamoxifen to adult mice carrying the *GFRα1-CreER*^*T2*^ or *Ngn3-CreER*^*TM*^ transgene, respectively, and a lineage reporter, *CAG-CAT-EGFP*, so that all descendants of these cells could be traced by GFP expression (hereafter designated GFRα1- or Ngn3-induced cells, respectively) (Figure 1B). Two days after labeling, single-cell suspensions prepared from donor testes were transplanted, without fractionation, into seminiferous tubules of adult host mice whose germ cells had been removed by busulfan treatment (Figure 1C). With a total of 10^6^ testicular cells transplanted per host testis, donor spermatogonia were seeded in a manner sparse enough to form separated clonal clusters. 2 days after transplantation of GFRα1- or Ngn3-induced cells, we observed 100 or 280 GFP^+^ cells, respectively, scattered over 1,700-mm-long tubules in each host testis (Figure 1F; Table S2). Because, starting at such low cell densities, merging of independently labeled cells is unlikely, spatially isolated clusters of GFP^+^ cells and single GFP^+^ cells were considered distinct “clones” (STAR Methods). Then, over timescales spanning post-transplantation day 2 (TP2) through TP180, the entire tubules that comprised each host testis were collected for whole-mount immunofluorescence (IF) (Figure 1D). All clusters of GFP^+^ donor cells in the entire specimen were counted as clones, regardless of whether they included cells attached to the basement membrane. Every clone was scored based on its constituent syncytial unit identity (A_s_, A_pr_, or A_al_) and differentiation status (GFRα1^+^ A_undiff_, GFRα1^–^ A_undiff_, and more advanced cells, identified as GFRα1^+^/promyelocytic leukemia zinc finger protein (Plzf)^+^, GFRα1^–^/Plzf^+^, and GFRα1^–^/Plzf^–^ cells, respectively), whereas no GFRα1^+^/Plzf^–^ cells were observed. However, in samples harvested on TP30 and later, when surviving clones were too large for all constituent GFP^+^ cells to be scored, GFRα1^+^ cell numbers and the spatial extent of the clone (i.e., colony length in the tubules) were quantified (Figures 1E and 1I; Tables S1A–S1H).

### Repopulation of donor-derived spermatogonia is accompanied by large-scale cell loss

**Figure 2:**
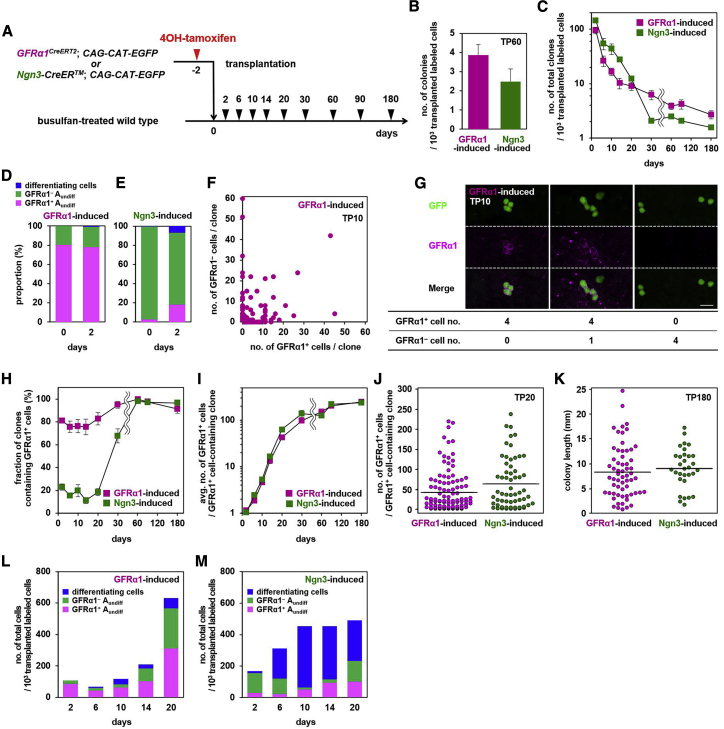
Post-transplantation dynamics of GFRα1- and Ngn3-induced cells (A) Schedule for TM injection (B–M). (B) Numbers of repopulating colonies observed 60 days after GFRα1- or Ngn3-induced cells were transplanted. Average ± SEM of the number of GFP^+^ colonies per 10^3^ transplanted labeled cells from 14 and 12 host testes for GFRα1- and Ngn3-induced donors, respectively, are shown. p = 0.11 (Student’s t test). (C) Total numbers of GFRα1- and Ngn3-induced clones observed in host tubules, normalized for 10^3^ transplanted labeled cells. (D and E) Compositions of GFRα1-induced (D) and Ngn3-induced (E) cells transplanted (TP0) and those found on TP2. (F) Composition of GFRα1^+^ and GFRα1^–^ cells in individual clones at TP10. (G) Examples of GFRα1-induced GFP^+^ clones at TP10, shown in (F), in whole-mount seminiferous tubules stained for GFP (green) and GFRa1 (magenta), scored as shown below. Scale bar, 25 μm. (H) Percentage of GFRα1- and Ngn3-induced clones with GFRα1^+^ content. (I) Average number of GFRα1^+^ cells per clone containing GFRα1^+^ cells originating from GFRα1- and Ngn3-induced cells. (J and K) Size distribution of clones derived from GFRα1- or Ngn3-induced cells: GFRα1^+^ cell number, TP20 (J) and colony length, and TP180 (K). (L and M) Numbers of GFRα1-induced (L) and Ngn3-induced (M) cells observed in host seminiferous tubules at the indicated time points, classified into GFRα1^+^ (pink) and GFRα1^–^ (green) A_undiff_ and differentiating cells (blue; containing Kit^+^ spermatogonia, spermatocytes, and spermatids). Values in (B), (C), (H), and (I) are shown as averages ± SEM; bars in (J) and (K) indicate the average.

Based on the clonal analysis of transplanted spermatogonia (Figure 2A), we found that GFRα1^+^ and Ngn3^+^ cells, each harboring repopulation potential (Garbuzov et al., 2018; Nakagawa et al., 2007), were capable of forming long-term repopulating colonies with comparable efficiency (Figure 2B). Most colony-forming activity was recovered from these cell fractions, reinforcing previous observations that the vast majority of repopulation activity resides in A_undiff_ (Ohbo et al., 2003; Shinohara et al., 2000; Figure S2B). However, for both fractions, although unexpectedly large numbers of cells were found to be able to settle in host tubules, the vast majority of resulting clones subsequently disappeared without forming repopulating colonies over the long term (Figure 2C).

The largest degree of clonal loss was observed during the first week following transplantation. Because SSCs take more than 1 month to fully differentiate and leave the tubules, and because the GFP label provides an indelible mark, we reasoned that clonal loss must be associated with cell death. In addition to cell death, multiple other events occur in parallel during this period. After attachment to the surface of Sertoli cells, a fraction of donor spermatogonia rapidly translocate to the basement membrane and start to proliferate, as evidenced by clones containing multiple cells at TP2 and TP6 (Tables S1A, S1B, and S1D). In contrast, other donor cells take longer to translocate or undergo cell death. By TP8, all surviving A_undiff_ were found on the basement membrane (Figures S1B–S1E). However, clonal loss continued beyond TP8, indicating that donor cells undergo cell death even after they have translocated onto the basement membrane and undergone cell proliferation (Figure 2C; Figure S1D). Subsequently, surviving clones expanded their territory, staying within the basal layer until TP20–TP30, and then cells underwent translocation with full reconstitution of spermatogenesis by TP60. At this time point, clones took the form of repopulating “colonies” (Figure 1I; Figures S1F–S1I), with the central region reaching a quasi-steady state and the periphery remaining “regenerative.” At the population level, the observed behavior of donor cells following transplantation and repopulation were consistent with the findings of previous studies (Nagai et al., 2012; Nagano et al., 1999; Nagano, 2003). However, contrary to the hypothesis advanced by Nagano, (2003), that most transplanted SSCs survive and remain quiescent until around TP10, our clonal-level fate analyses revealed that proliferation and death of donor cells start shortly after transplantation.

### Donor spermatogonia follow divergent clonal fates in host testes

To gain deeper insight into the pattern of individual cell fate decisions during the repopulation process, we conducted an in-depth investigation of the clonal fate of GFRα1- and Ngn3-induced donor cells based on their gene expression status. Upon transplantation, ~80% of GFRα1-induced cells were GFRα1^+^, and this proportion was maintained in cells observed in host tubules on TP2 (Figure 2D). In contrast, although nearly all transplanted Ngn3-induced cells were GFRα1^–^ A_undiff_, ~20% of those observed in host testes on TP2 were GFRα1^+^ (Figure 2E). These observations indicate that, following transplantation, GFRα1^+^ and GFRα1^–^ A_undiff_ are capable of settling in the tissue and, second, that a considerable level of conversion of cells from a differentiation-primed GFRα1^–^ state to a GFRα1^+^ state, a process we refer to as “reversion” (Nakagawa et al., 2010), occurs shortly after transplantation. Because comparable numbers of GFRα1- and Ngn3-induced cells were found in host tubules on TP2 (Figure 2C), we reasoned that selective settlement of GFRα1^+^ cells was unlikely.

Following transplantation, we expected that stem cells would exclusively self-renew rather than differentiate in the “empty” niche microenvironment. However, we found that surviving clones were comprised of variable numbers of GFRα1^+^ and GFRα1^–^ cells from the earliest time points (Figure 2F; Figures S2C–S2G). Although some clones contained only GFRα1^+^ cells, others also contained GFRα1^–^ cells. Clones without GFRα1^+^ cells were also observed, comprising some 15%–30% and 80%–90% of the total GFRα1- and Ngn3-induced donor clones, respectively; these figures remained stable over the first month after transplantation (Figures 2F–2H). Some of these clones contained no A_undiff_ but comprised differentiating spermatogonia, spermatocytes, and/or spermatids (collectively designated “differentiating cells” hereafter) only. Such “differentiating clones,” many of which derived from Ngn3-induced donor cells, declined in number after TP14 (Figure S2H). Given that the entire differentiation process takes about 1 month, we reasoned that this decline must be due to cell death, suggesting that differentiating germ cells are prone to die under such low-density conditions. However, a few differentiating clones survived, enlarged, and differentiated on schedule; consistently, on TP30, clones containing only elongating spermatids were observed (Figures S2H–S2J). Therefore, following transplantation, a considerable fraction of donor spermatogonia (Ngn3-induced cells in particular) undergo synchronous proliferation and differentiation without self-renewal, although their colonies were too small to support robust spermatogenesis. Beyond the first month after transplantation, the vast majority of surviving clones contained at least one GFRα1^+^ cell, and clonal loss was observed at much lower frequencies (Figures 2C and 2H).

All clones that persisted after 1 month following transplantation were found to harbor a population of A_undiff_ that contain one or more GFRα1^+^ cells, regardless of their cell of origin (GFRα1^+^ or Ngn3^+^ cell-derived). It therefore follows that, for Ngn3-induced donor spermatogonia to establish repopulating colonies, the transplanted cell, or at least one of its progenies, must convert to a GFRα1^+^ state (Nakagawa et al., 2007, 2010). When we focused only on clones containing one or more GFRα1^+^ cells, a highly similar average and distribution of clone size (as assessed by the number of GFRα1^+^ cells or, at later time points, the length of repopulating colonies within tubules) as well as survival probability were found at all time points, regardless of whether they originated from GFRα1- or Ngn3-induced cells (Figures 2I–2K; Figures S2K–S2P). This suggests that GFRα1^+^ cells converted from a Ngn3^+^ state following transplantation exhibit long-term behavior indistinguishable from that of *bona fide* GFRα1^+^ cells.

Finally, following transplantation of testicular cells prepared from donor mice ubiquitously expressing GFP, by TP180, all persisting colonies were found to harbor GFRα1^+^/RARγ^–^, GFRα1^+^/RARγ^+^, and GFRα1^–^/RARγ^+^ subpopulations of A_undiff_ in similar proportions, typically in larger clones (Figures S2Q–S2V; Table S3). Given that GFRα1^+^ and Ngn3^+^ cells each contribute to roughly one half of the colony-forming activity of the total testicular cells (Figure 2B; Figure S2B), this observation supports the conclusion above, indicating that all transplantable stem cells can reconstitute the heterogeneous composition of A_undiff_ cells regardless of their original state.

### Population-level dynamics of donor spermatogonia provide evidence of convergent fates

To quantify the changes in donor cell state composition following transplantation, we analyzed the population averages of cell states from TP2–TP20, when clones were small enough to individually score all constituent cells (Figures 2L and 2M). For the GFRα1-induced population, the total number of donor cells was found to be roughly stable until TP10. However, this behavior was not a result of cellular quiescence but reflects a dynamic process where cell proliferation and death roughly compensate, as described earlier (Figures 2C and 2I). From TP10, the number of A_undiff_ (GFRα1^+^ and GFRα1^–^) start to increase. On TP10, a small but considerable number of differentiating cells (i.e., differentiating spermatogonia and beyond) emerged that were most likely derived from the minority population of GFRa1^–^ A_undiff_ observed on TP2 (Figure 2D). Subsequently, the gross number of differentiating cells increased moderately based on cell proliferation and supply from A_undiff_, whereas they also undergo cell death (Figure 2C; Figure S2H).

For Ngn3-induced cells, the major fraction (~80%) of cells on TP2 were found to be GFRα1^–^ (Figure 2E). On TP10, many of these cells had disappeared, whereas many differentiating cells appeared, suggesting that the bulk of GFRα1^–^ A_undiff_ proceed to differentiate (Figure 2M). The number of differentiating cells remained largely constant, as observed for GFRα1-induced cells. Moreover, we found that the minority population of GFRα1^+^ cells observed on TP2 followed kinetics similar to those observed for GFRα1-induced cells (Figures 2L and 2M). After a largely stable period, the total number of GFRα1^+^ cells increased from TP10 on, followed by an increase of GFRα1^–^ A_undiff_ to a degree comparable with GFRα1^+^ cells by TP20. Such consistency of the population-level cell dynamics reinforces the conclusion drawn from the long-term clonal analyses: GFRα1^+^ cells converted from the Ngn3^+^ state function as *bona fide* GFRα1^+^ cells following transplantation.

### Transplanted spermatogonia interconvert between singly isolated and syncytial states

**Figure 3:**
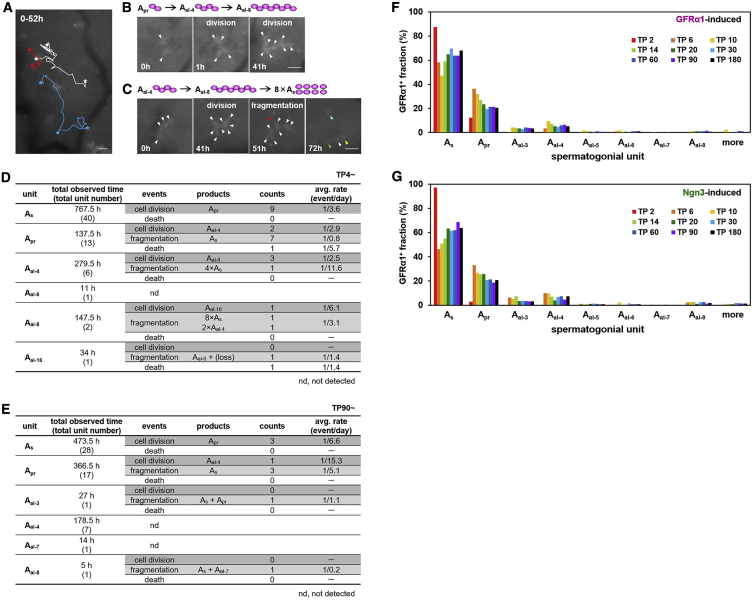
Intravital live imaging of transplanted A_undiff_ and their syncytial composition (A) Trajectories of three GFRα1^+^ A_undiff_ over 52 h of observation in intravital live imaging of a host mouse testis (Video S1), shown in different colors. Dots and stars indicate the starting and ending points, respectively. Bifurcations indicate cell division. (B) Selected frames of a filmed example of A_pr_ → A_al4_ → A_al8_ serial incomplete divisions (Video S2). (C) An example of A_al4_ → A_al8_ incomplete division followed by fragmentation into 8 A_s_ (Video S3). Scale bars, 25 μm. (D and E) Summary of cell division, fragmentation, and death of GFRα1^+^ A_undiff_ observed in intravital live imaging of host mouse testes at TP4 (D) and TP90 (E) after testicular cells of *GFRα1*^*EGFP*^ mice were transplanted. Average rates of these events were calculated as counts of observed events/total observation time. (F and G) Length distribution of the GFRα1^+^ syncytial units of GFRα1-induced (F) and Ngn3-induced (G) donor cells following transplantation over time, summarized from data in Tables S1A, S1C, and S1E for (F) and Tables S1A and S1E for (G), respectively.

To further elucidate the diverse fate behaviors of transplanted spermatogonia, we performed intravital live-imaging analysis in host testes. Previous studies of the behavior of GFRα1^+^ (*Gfrα1*-GFP^+^) cells during normal homeostasis showed that these cells migrate actively over the basement membrane while interconverting between singly isolated and syncytial states through incomplete divisions and fragmentation via intercellular bridge breakdown (Hara et al., 2014). Using the same live-imaging approach, we observed that, shortly after transplantation (TP4–TP6), donor *Gfrα1*-GFP^+^ cells were highly migratory and showed incomplete division and syncytial fragmentation, similar to that seen in homeostasis, albeit with higher rates (Figures 3A–3D; Videos S1, S2, and S3). Consistent with these observations, by reanalyzing the clonal fate data for transplanted GFRα1- and Ngn3-induced cells (Tables S1A, S1C, and S1E), we found that donor cell syncytia were observed as early as TP2 and that the composition of syncytial lengths of GFRα1^+^ cells converged quickly onto the long-term, steady-state distribution (Figures 3F and 3G). We also observed frequent cell death, an event seen rarely in homeostasis (Figure 3D; Hara et al., 2014), consistent with early clonal loss (Figure 2C). These observations indicated that, following transplantation, instead of undergoing complete division in the uncrowded open niche environment, donor spermatogonia constantly interconvert between singly isolated and syncytial states while showing frequent cell death.

Video S1. Active migration of GFRα1+ spermatogonia observed in intravital live imaging, filmed for 2 days from TP4, related to Figure 3A Elapsed time is shown in day:hour:minute.4

Video S2. Sequential incomplete divisions of GFRα1+ spermatogonia observed in intravital live imaging, filmed for 4 days from TP4, related to Figure 3B Elapsed time is shown in day:hour:minute.5

Video S3. Fragmentation of interconnected GFRα1+ spermatogonia observed in intravital live imaging, filmed for 4 days from TP4, related to Figure 3C Elapsed time is shown in day:hour:minute.6

On TP90, when the donor cells have established repopulating colonies, these rates were found to be similar to those observed in undisturbed testes harboring homeostatic spermatogenesis (Figure 3E; Hara et al., 2014), supporting evidence, based on histology, that homeostasis in repopulating colonies is restored.

### Stochastic fate behavior of the equipotent donor cell population during repopulation

Based on the observations above, we next questioned the origin of the divergent fate behavior during repopulation after transplantation. Here we noted that donor SSC behavior showed a number of striking similarities with the dynamics of SSCs during homeostasis (Hara et al., 2014; Klein et al., 2010; Nakagawa et al., 2007). In particular, under both conditions, clones showed a high degree of variability in fate behavior, with some clones expanding and persisting while others became “extinct” over the short term. Moreover, under both conditions, such clonal behavior was associated with active migration and interconversion between singly isolated and syncytial morphologies. Previously, it has been shown that, in homeostasis, such intricate SSC clone dynamics can be predicted quantitatively using a minimal modeling scheme (Hara et al., 2014; Klein et al., 2010). In this model, to mimic the homogeneous coarse-grained density of SSCs over the tubules, GFRα1^+^ spermatogonia were embedded on a cylindrical lattice, with the occupancy of each site limited to precisely one GFRα1^+^ spermatogonial “unit” (either a singly isolated cell or syncytium). Within this framework, GFRα1^+^ units were allowed to undergo rounds of incomplete cell division, syncytial fragmentation, and differentiation with defined, unchanging probabilities. Then, to ensure density homeostasis, loss of a GFRα1^+^ unit through differentiation was perfectly compensated by the “duplication” (i.e., fragmentation) of a GFRα1^+^ unit on a neighboring lattice site. Despite its simplicity, this modeling scheme has been shown to have a strong predictive capacity in homeostasis, quantitatively capturing the range of observed clone fate behaviors, supporting the conclusion that all GFRα1^+^ cells follow the same probabilistic rules.

**Figure 4:**
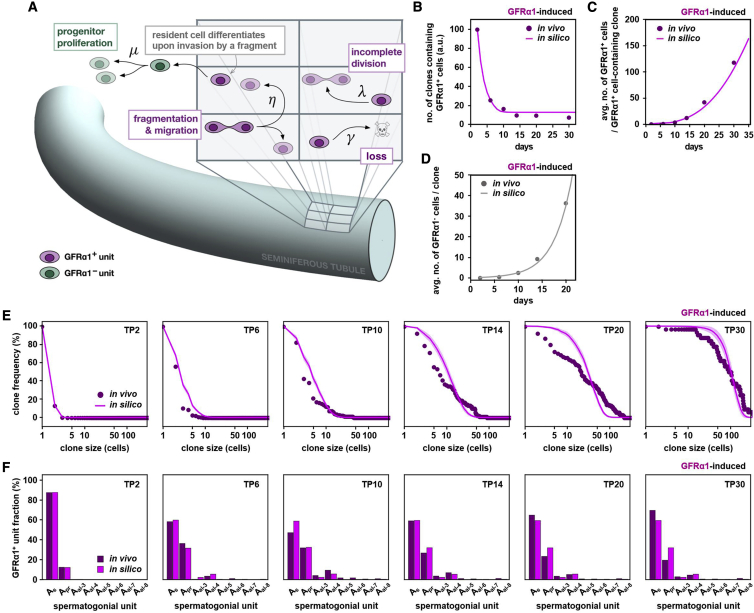
Modeling scheme and prediction of post-transplantation fate behavior of GFRα1-induced donor cells (A) Schematic of the biophysical model. Tubules are represented as lattices, each site hosting one GFRα1^+^ unit, that undergo incomplete division and fragmentation (and loss during an initial post-TP phase), with rate parameters as indicated in Table S4. See STAR Methods for details. (B–D) Model predictions of the number of clones with GFRα1^+^ content relative to TP2 (B), their average number of GFRα1^+^ cells (C), and average number of GFRα1^–^ cells per clone (D) after GFRα1-induced cells were transplanted (dots, experiments; curve, model). (E) Cumulative clone size distribution of GFRα1^+^ cells among persisting clones derived from GFRα1-induced donor cells (dots, experiments; curve, model). (F) Composition of GFRα1^+^ syncytia over time, averaged over persisting clones after GFRα1-induced cells were transplanted (dark, the experiment shown in Figure 3F; bright, model).

We therefore wanted to find out whether SSC dynamics post-transplantation could be predicted by the same modeling scheme after appropriate adjustment to account for the non-steady-state nature of the repopulation process. In particular, we questioned whether the observed variable clonal fate behavior could be explained by common statistical rules, pointing at the equipotency of the SSC population. We sought to define a modeling scheme based on a minimal set of parameters (Figure 4A; Table S4; STAR Methods). As a starting condition, to reflect the situation immediately following transplantation, seminiferous tubules (i.e., the lattice) were seeded sparsely with singly isolated GFRα1^+^ cells. Then, as described previously for homeostatic conditions, cells were allowed to undergo incomplete division, syncytial fragmentation, and differentiation. Here we imposed much higher rates of incomplete cell division and syncytial fragmentation compared with homeostatic conditions, with estimates based on measurements using intravital live imaging from TP4 (Figure 3D; Hara et al., 2014). In addition, to capture the highly elevated rates of cell death during the early phase of repopulation, GFRα1^+^ cells were presumed to undergo cell death at a constant rate over an initial period post-transplantation, whereas any residual effects of cell death at longer times were neglected. To further simplify the model, the behavior of GFRα1^–^ cells (including GFRα1^–^ A_undiff_ and more advanced Kit^+^ spermatogonia, spermatocytes, and haploid cells) was encapsulated by an effective proliferation rate, accounting for the net outcome of cell proliferation and death. Further, we did not explicitly include the process of reverse transitioning from GFRα1^–^ to GFRα1^+^ cells or differentiation uncompensated by the fragmentation of neighboring SSC units, processes that may also occur following transplantation in the empty niche environment. However, the positive and negative effects of these events on the number of GFRα1^+^ and GFRα1^–^ cells are included implicitly in the effective rates of cell death of GFRα1^+^ cells and proliferation of GFRα1^–^ cells (STAR Methods).

Then, using the measured rate of incomplete division based on live imaging and adjusting just four fit parameters (the effective rates of syncytial fragmentation and cell loss, the period over which cell loss occurs for the GFRα1^+^ compartment, and the effective proliferation rate of GFRα1^–^ cells), we found that the model could quantitatively capture the wide range of clone behaviors for GFRα1- and Ngn3-induced donor cells from TP2 through to TP30. These include the clone survival rate (i.e., the probability that a clone maintained one or more GFRα1^+^ cells; Figure 4B; Figure S3A), the average and distribution of clone size (as scored by the constituent number of GFRα1^+^ cells; Figures 4C and 4E; Figures S3B and S3D), the length composition of syncytial units in the GFRα1^+^ compartment (Figure 4F; Figure S3E), and production of GFRα1^–^ cells (Figure 4D; Figure S3C), which were all predicted by the model with high accuracy (Figure S3F–S3H; STAR Methods). Such a high predictive capacity of the model, despite its simplicity, lead to the conclusion that the divergent clonal fates of donor cells following transplantation may simply be a consequence of stochastic fate selection of equipotent SSCs rather than due to innate heterogeneity in the potency of SSCs or local niche regulation.

### Suppression of RA synthesis enhanced the repopulation efficiency

**Figure 5:**
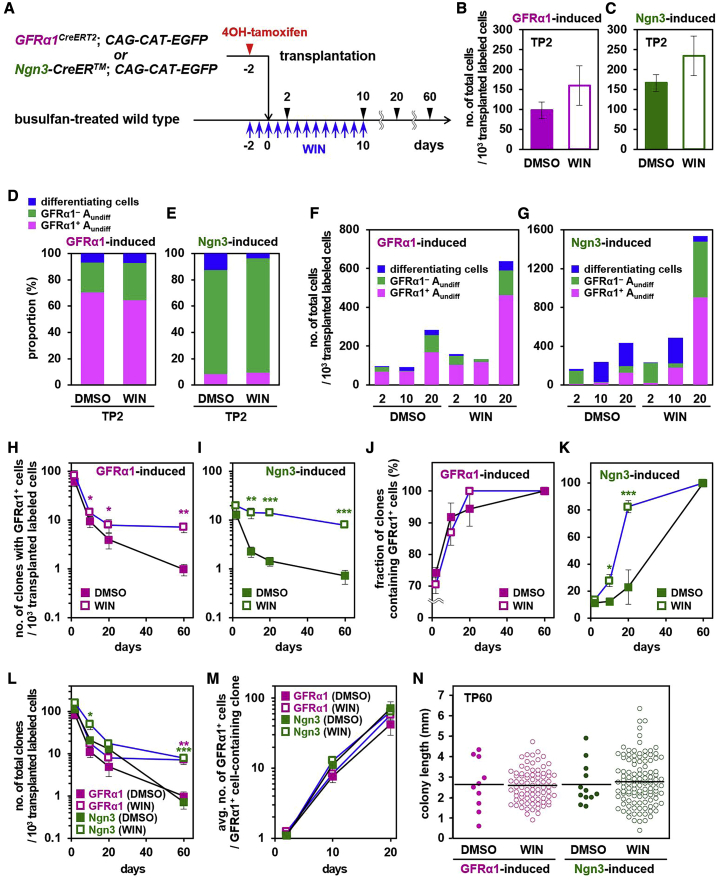
Enhanced repopulation caused by temporary block of RA synthesis with WIN (A) The experimental schedule. (B–E) Numbers (B and C) and differentiation status (D and E) of GFRα1- and Ngn3-induced donor cells on TP2 with DMSO (controls) or WIN treatment. (F and G) Numbers of GFRα1-induced (F) and Ngn3-induced (G) cells observed in host seminiferous tubules, with DMSO (controls) or WIN treatment at the indicated time points, classified into GFRα1^+^ (pink) and GFRα1^–^ (green) A_undiff_ and differentiating cells (blue). (H–K) Percentages (H and I) and numbers (J and K) of clones including GFRα1^+^ cells, derived from GFRα1- or Ngn3-induced cells transplanted into the host with DMSO (controls) or WIN treatment. (L) Total number of clones derived from GFRα1- and Ngn3-induced donor cells in hosts treated with DMSO (controls) or WIN. (M) Average number of GFRα1^+^ cells per clone containing GFRα1^+^ cells, originating from GFRα1- and Ngn3-induced cells in hosts treated with DMSO (controls) or WIN. (N) Lengths of repopulating colonies derived from GFRα1- and Ngn3-induced donor cells on TP60 with DMSO (controls) or WIN treatment. Values in (B), (C), and (H)–(M) are shown as averages ± SEM; bars in (N) indicate averages. ^∗^p < 0.05, ^∗∗^p < 0.01^∗∗^, and ^∗∗∗^p < 0.001 between DMSO- and WIN-treated hosts.

Motivated by the conclusions of the modeling analysis, we then tested whether the repopulation efficiency could be increased by tuning donor cell fates in host tissue. We reasoned that blocking the differentiation of donor cells during the initial active phase could expand the size of the founder SSC population, leading to an increased probability to persist long term. In particular, to block differentiation of Ngn3^+^ to Kit^+^ differentiating spermatogonia, a process driven by RA signaling (Figure 1A), we turned to WIN18,446 (WIN). WIN is a useful chemical inhibitor of RA synthesis in the testis without significant off-target effects; its capacity *in vivo* to block differentiation of innate spermatogonia has been demonstrated in developing and mature testes (Agrimson et al., 2017; Amory et al., 2011; Busada et al., 2015; Paik et al., 2014). Based on this idea, host mice were treated with WIN once per day from TP(−2) to TP10. Again, to follow the clonal fate, GFRα1- and Ngn3-induced donor cell suspensions were used for transplantation, and GFP^+^ donor cells were scored based on their constituent syncytial unit length (A_s_, A_pr_, and A_al_) and gene expression status (Figure 5A; Tables S1G, S1H, and S2). Here, A_undiff_ were identified as cells expressing GFRα1, RARγ, or both (including GFRα1^+^/RARγ^–^, GFRα1^+^/RARγ^+^, and GFRα1^–^/RARγ^+^), whereas GFRα1^–^/RARγ^–^ cells were designated as differentiating. RARγ was used because it acts predominantly in mediating the differentiation-promoting RA signal in these cells (Gely-Pernot et al., 2012; Ikami et al., 2015). Its expression status therefore provides cues to interpret the mechanism of WIN action. On TP2, WIN treatment did not significantly change the numbers of donor cells found in host tubules or their gene expression profiles compared with DMSO-treated controls (Figures 5B–5E).

We first analyzed donor cell fate at the population level to evaluate the effect of WIN on the differentiation status. On TP10, emergence of differentiating cells from GFRα1-induced donor cells was suppressed by WIN treatment; all donor cells were in the A_undiff_ compartment (Figure 5F). For Ngn3-induced donor cells, although many differentiating cells were found on TP10 in the presence of WIN, these cells were synaptonemal complex protein 3 (SYCP3)^+^ spermatocytes, and no Kit^+^ differentiating spermatogonia were found, unlike with the DMSO-treated control (Figure 5G; Figure S4A). Consistent with this, the number of A_undiff_ was increased greatly by WIN (Figure 5G). These results indicate that WIN efficiently blocks differentiation of A_undiff_, although a significant fraction of Ngn3-induced cells was already beyond the WIN-sensitive (i.e., RA-dependent) differentiation step when transplanted. Interestingly, although WIN blocked differentiation of GFRα1^–^ (largely RARγ^+^) A_undiff_, we observed an increase of GFRα1^+^ cells, indicative of enhanced reverse conversion of GFRα1^–^ cells to a GFRα1^+^ state. This conclusion was further supported by the increase of GFRα1^+^/RARγ^+^ cells, which may represent a transitory state (Figures S4B and S4C). On TP20, for GFRα1- and Ngn3-induced donor cells, the effects of WIN treatment were more apparent, showing greater numbers of A_undiff_ than controls (Figures 5F and 5G). These results showed that WIN treatment during the initial phase after transplantation expands the pool of A_undiff_ by inhibiting their differentiation.

At the clonal level, WIN treatment had a significant effect on Ngn3-induced cell-derived clones; clones containing GFRα1^+^ cells and composed only of A_undiff_ were increased on TP10 and TP20 compared with the control (Figures 5I and 5K; Figure S4D). Conversely, the number of differentiating clones (without A_undiff_) was decreased on TP20 (Figure S4E). For GFRα1-induced donor cell-derived clones, in which the proportion of A_undiff_ was already large even without WIN treatment, changes induced by WIN treatment were proportionately smaller or statistically insignificant (Figures 5H and 5J; Figures S4D and S4E). However, as a long-term consequence, on TP60, the number of clones containing GFRα1^+^ cells showed an order of magnitude increase following WIN treatment for GFRα1- and Ngn3-induced donor cell-derived clones (Figures 5H and 5I). Large increases were also observed in the total number of surviving clones, further supporting the conclusion that a clone becomes stable when it has gained GFRα1^+^ cells (Figure 5L). Interestingly, in contrast to the increase in surviving clone numbers, enlargement of individual clones was not observed in terms of GFRα1^+^ cell numbers per clone or the spatial extent of resultant repopulating colonies in the long term on TP60 (Figures 5M and 5N). Thus, expansion of the undifferentiated cell pool mediated by WIN treatment effectively elevates the probability of each clone persisting over the long term, forming a repopulating colony rather than upsizing individual clones. These results further reinforce the conclusion that, from the extended equipotent population of SSCs, only a small fraction repopulate over the long term through stochastic cell fate selection.

### Fertility of the host was restored by WIN treatment on transplantation

**Figure 6:**
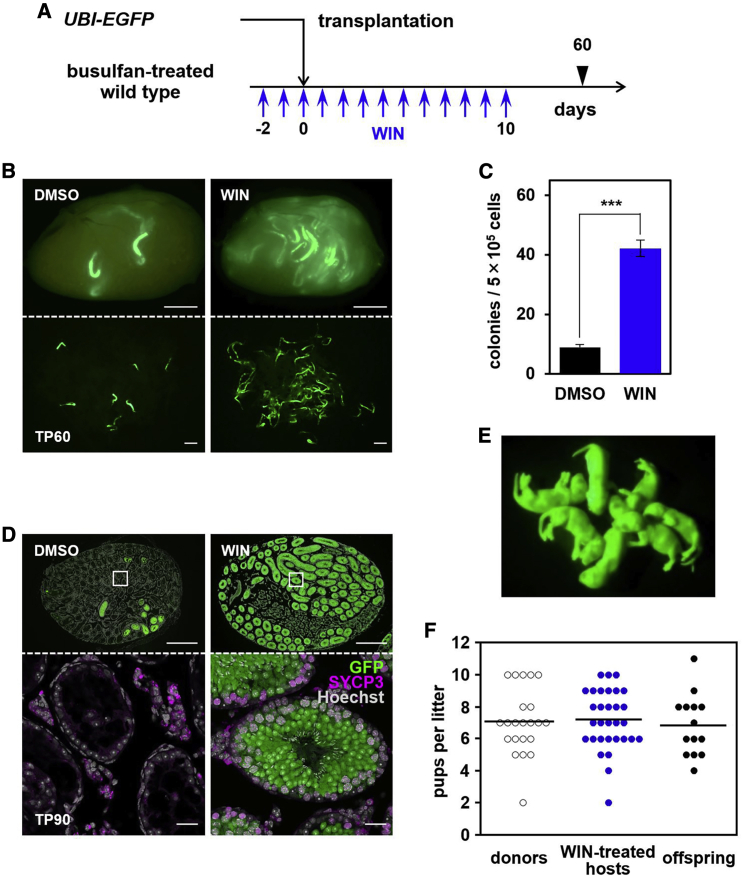
Fertility restoration by WIN treatment upon transplantation (A) Schedule to test the WIN effect on overall repopulation using *UBI-EGFP* donors. (B and C) Typical appearances of the host testes (top) and untangled seminiferous tubules (bottom) under a fluorescence stereomicroscope (B) and numbers of GFP^+^ colonies (C) on TP60 with DMSO (control) or WIN treatment. In (C), averages ± SEM from 16 (DMSO) and 15 (WIN) host testes are shown (^∗∗∗^p = 9.6E−17). (D) Representative cross-section of a host testis with DMSO (control) or WIN treatment on TP90, stained for GFP, SYCP3, and DNA (Hoechst 33342), with boxed regions enlarged below. (E) Viable GFP^+^ progeny obtained from a WIN-treated host (WIN-7 in Table S5) through natural mating. (F) Litter size from *UBI*-*EGFP* mice, WIN-treated hosts, and their offspring (5, 5, and 3 mice, respectively) after natural mating. DMSO-treated hosts produced no offspring. Bars indicate average values. Scale bars indicate 1 mm (B), 500 μm (D, top panel), and 25 μm (D, bottom panel).

Finally, we evaluated the effect of WIN treatment on overall repopulation efficiency, host fertility, and the next generation (Figure 6). Using *UBI-EGFP* mice showing ubiquitous GFP expression as donors, we observed a large increase in the number of long-term repopulating colonies in WIN-treated hosts (Figures 6A–6C). Transplantation of an unfractionated testicular cell suspension led to reconstitution of robust spermatogenesis over a major portion of seminiferous tubules of WIN-treated host testes, in stark contrast to DMSO-treated control hosts (Figure 6D). Consequently, 5 of 12 WIN-treated hosts sired donor-cell derived (GFP^+^) offspring with normal litter size through natural mating with normal female mice. In contrast, DMSO-treated control recipients produced no offspring (Figure 6E; Table S5). Moreover, offspring derived from WIN-treated host males showed normal growth and fertility (Figure 6F). Based on these observations, we concluded that sperm quality and next-generation individuals are not affected when host mice are treated with WIN or when otherwise differentiating cells (e.g., Ngn3^+^ A_undiff_) are recruited artificially to the self-renewing pool and contribute to long-term repopulation of spermatogenesis.

## Discussion

The ability to reconstitute the structure and function of lost tissue following transplantation has been recognized as one of the key properties of adult stem cells. Indeed, following early pioneering studies in hematopoiesis, post-transplantation restoration of tissue function was considered by many a gold standard method to define stem cell identity (Becker et al., 1963). Using such transplantation assays, stem cell potential has been assessed based on the long-term outcome; e.g., the donor contribution to reconstituted tissues. In mouse spermatogenesis, the identity and potential of SSCs have been assayed based on the number of repopulating colonies that form 2–3 months following transplantation (Brinster, 2002). Although this assay enables the “relative potential” of SCCs to be measured between different cell populations, very little has been known about the fate behavior underlying colony formation in host seminiferous tubules, hindering the ability to define SSC identity, their repopulation potential, and their absolute numbers.

In this study, we analyzed the fate of individual donor SSC-derived clones over an extended time course at single-cell resolution. We found that repopulation by donor spermatogonia in host tubules is not mediated by a definitive minority population of potent stem cells. Rather, our results show that a greater number of cells in the A_undiff_ population harbor repopulation potential compared with the number that actually repopulate in the long term. Although many donor cells are initially able to settle and divide in tissue, through chance fate decisions, only a minority of resulting clones expand, persist, and form repopulating colonies, whereas the majority disappear as a result of stochastic differentiation and/or cell death of their self-renewing progenies.

GFRα1^+^ and Ngn3^+^ cells, which largely self-renew and transit amplify, respectively, under homeostatic conditions, are capable of founding repopulating colonies (Carrieri et al., 2017; Nakagawa et al., 2007; Nakagawa et al., 2010). The results obtained in this study suggest that differences in the observed efficiencies with which GFRα1^+^ and Ngn3^+^ fractions repopulate is simply a reflection of their short-term potential to maintain or acquire GFRα1^+^ state identity following transplantation. This finding highlights the importance of conversion from GFRα1^–^ (largely Ngn3^+^) to GFRα1^+^ states, or reversion, in facilitating the repopulation process (Nakagawa et al., 2010). However, when donor cells or their progenies acquire the GFRα1^+^ state identity, their repopulation potential does not differ in the long term, irrespective of their original state.

Importantly, our findings show that repopulation potential is not invested in a subset of SSCs but shared equally and broadly over an extended population of transplanted cells, regardless of whether their progenies actually persist over the long term. This conclusion was supported by the fact that the highly intricate fate dynamics of donor cell-derived clones can be predicted quantitatively by a minimal modeling scheme in which all SSCs follow the same probabilistic rules of fate selection and consolidated experimentally using WIN treatment. This model is based on minimal adjustment of the framework used to describe SSC dynamics in homeostasis (Hara et al., 2014; Klein et al., 2010). Therefore, independent of tissue context (homeostatic or regenerative), SSCs select their fate according to common principles of stochasticity so that only a fraction of cells, by chance, persist over the long term. These findings emphasize that, according to the same fate rules, differing degrees of constraint (e.g., SSC density) may result in distinctive context-related dynamics. Recent studies have proposed that, under homeostatic conditions, SSC dynamics result from a feedback mechanism based on competition for fate determinants or mitogens (Kitadate et al., 2019; Jörg et al., 2021). The current findings indicate that the same mechanism might also underpin the regenerative dynamics of donor SSCs after transplantation. A similar class of models captures the nature of homeostatic stem cell dynamics across a wide range of tissue types (Klein and Simons, 2011), raising the interesting question of whether a similar biophysical framework can describe the regenerative dynamics of cell populations in other tissues.

Our experiments using WIN have served to strengthen the model hypothesis that stem cell potential is not limited to a distinct subfraction of cells but shared across a broader population of A_undiff_, most of which are lost by chance through differentiation and cell death after initial settlement. Based on this observation, we established WIN treatment as a strategy that greatly enhances long-term repopulation efficiency by tuning the fate of donor spermatogonia. By combining transplantation with simultaneous administration of WIN, many donor SSC clones that would otherwise have been lost are able to contribute to long-term repopulation. Crucially, the resultant sperm originating from these otherwise vanishing clones were shown to have normal function, generating fertile offspring.

Multiple factors could contribute to the increase of repopulation efficiency by WIN treatment, whose primary action is to reduce the tissue concentration of RA by inhibiting alcohol dehydrogenase activity (Amory et al., 2011). Given that RA induces differentiation of a fraction of A_undiff_ expressing Rarγ^+^, a key RAR, in a cell-autonomous fashion, we reason that the primary action of WIN treatment is to inhibit differentiation of RARγ^+^ A_undiff_ (largely Ngn3^+^ and GFRα1^–^), leading to expansion of the GFRα1^+^ SSC pool via conversion of GFRα1^–^/Rarγ+ cells when not exposed to RA (Endo et al., 2017; Gely-Pernot et al., 2012; Hogarth et al., 2011; Ikami et al., 2015). In addition, because RA also affects gene expression of Sertoli cells and perhaps other somatic cells (Sugimoto et al., 2012), WIN treatment may also function indirectly to generate a microenvironment that is not conducive for spermatogonial differentiation.

From the perspective of practical application, here we developed a robust strategy to improve post-transplantation repopulation efficiency using WIN, a useful RA synthesis inhibitor capable of blocking spermatogonial differentiation *in vivo* (Agrimson et al., 2017; Hogarth et al., 2013). Crucially, successful generation of offspring through natural mating of “surrogate fathers” without intracytoplasmic sperm injection (ICSI), transplanted with unfractionated testicular cell suspension (rather than enriched fractions or *in-vitro-*expanded cells), and using adult donors and hosts (rather than immature, prepubertal animals) will significantly improve the translational potential of this method. Moreover, the reversible and tissue-specific action of WIN in multiple species promises a wide range of practical applications (Amory et al., 2011; Heller et al., 1961).

Based on the success of this approach, further strategies to improve repopulation efficiency can be envisaged, including promotion of reverse transitioning from a GFRα1^–^ to a GFRα1^+^ cell state, promotion of self-renewal of GFRα1^+^ cells, and suppression of cell death. Their successful implementation would open up a broader range of application of SSC transplantation technology, from restoration of fertility in young male individuals with cancer following therapy (Firlej et al., 2012) to preservation of genetic diversity of farm animals or endangered species (Honaramooz and Yang, 2010).

### Limitations of study

The minimal modeling scheme used to analyze the data makes the simplifying assumption that SSC function following transplantation takes place in a spatially homogeneous environment. Although this model has been shown to have a high predictive capacity, emphasizing the existence of a simple stochastic principle underlying SSC fate behavior, the actual host environment includes some degree of heterogeneity spatially (related to the extratubular interstitium/vasculature) and temporally (related to the periodic seminiferous epithelial cycle) (Yoshida, 2018), which is likely to affect SSC behavior. Similarly, the modeling analysis captures only the effective or “gross” rate of donor cell differentiation (GFRα1^+^ to GFRα1^–^) but not the separate net differentiation and reverse transition rates (GFRα1^–^ to GFRα1^+^). It would deepen our understanding of the transplantation process and further improve strategies to increase the repopulation efficiency if such heterogeneities and detailed SSC state transitions would be investigated and modulated.

## STAR★Methods

### Key resources table

**Table:** 

REAGENT or RESOURCE	SOURCE	IDENTIFIER
**Antibodies**		

Rat monoclonal anti-GFP (used at 1:400)	Nacalai Tesque	Cat# 04404-84; RRID: AB_10013361
Goat polyclonal anti-GFRα1 (used at 1:500)	R&D systems	Cat# AF560; RRID: AB_2110307
Rabbit monoclonal anti-RARγ (used at 1:200)	Cell Signaling	Cat# 8965; RRID: AB_10998934
Rabbit polyclonal anti-Plzf (used at 1:200)	Santa Cruz	Cat# sc-22839; RRID: AB_2304760
Goat polyclonal anti-c-Kit (used at 1:500)	R&D systems	Cat# AF1356; RRID: AB_354750
Mouse monoclonal anti-SYCP3 (used at 1:500)	Abcam	Cat# ab97672; RRID: AB_10678841
Rabbit polyclonal anti-GFP (used at 1:300)	Thermo Fisher	Cat# A-11122; RRID: AB_221569
Chicken polyclonal anti-GFP (used at 1:300)	Abcam	Cat# Ab13970; RRID: AB_300798
Mouse monoclonal anti-Plzf (used at 1:100)	EMD Millipore	Cat# OP128; RRID: AB_2218935
Rabbit polyclonal anti-Collagen IV (1:500)	Abcam	Cat# ab6586; RRID: AB_305584
Rat monoclonal anti-Mouse E-cadherin (1:1,000)	Takara Bio	Cat# M108

**Chemicals, peptides, and recombinant proteins**		

4-Hydroxytamoxifen	Sigma-Aldrich	Cat# H7904
Busulfan (1,4-butanediol dimethanesulfonate)	Wako	Cat# B7973
WIN18,446	Cayman Chemical	Cat# 14018
dimethyl sulfoxide	Nacalai Tesque	Cat# 09659-14
Hoechst 33342	Thermo Fisher	Cat# H3570
Fluoro-KEEPER Antifade Reagent	Nacalai Tesque	Cat# 12593-64
Type IV Collagenase	Sigma-Aldrich	Cat# C5138
DNase I	Sigma-Aldrich	Cat# D5025
Type I-S Hyaluronidase	Sigma-Aldrich	Cat# H3506

**Experimental models: Organisms/strains**		

Mouse: *GFRα1*^*CreERT2*^: C57BL6J	Uesaka et al., 2007	N/A
Mouse: *Ngn3-CreER*^*TM*^: C57BL6J	Yoshida et al., 2006	N/A
Mouse: *CAG-CAT-EGFP*: C57BL6J	Kawamoto et al., 2000	N/A
Mouse: *GFRα1*^*EGFP*^: C57BL6J	Uesaka et al., 2007	N/A
Mouse: *Nanos3*^*Cre*^: C57BL6J	Suzuki et al., 2008	N/A
Mouse: *UBI-GFP*: C57BL6J	The Jackson Laboratory	Cat# 004353
Mouse: *CAG-EGFP*: C57BL6J	Japan SLC	N/A
Mouse: *Kit*^*W/Wv*^: WBB6F1	Japan SLC	N/A
Mouse: *Ngn3-EGFP*: C57BL6J	(Yoshida et al., 2004)	N/A

**Software and algorithms**		

Metamorph software	Molecular Devices	https://www.moleculardevices.com/
CellSens Standard software	Olympus	https://www.olympus-lifescience.com/en/software/cellsens/image-processing-and-sharing/

### Resource availability

#### Lead contact

Further information and requests for resources should be directed to and will be fulfilled by the lead contact, Shosei Yoshida (shosei@nibb.ac.jp).

#### Materials availability

This study did not generate new unique reagents.

#### Data and code availability

All data are available in the main text or the supplementary materials. The code used for simulating clonal fatescan be found on GitHub (https://github.com/BenSimonsLab/Nakamura_Cell-Stem-Cell_2021).

### Experimental model and subject details

#### Animals

*GFRα1*^*CreERT2*^ (Uesaka et al., 2007), *Ngn3-CreER*^*TM*^ (Yoshida et al., 2006), *CAG-CAT-EGFP* (Kawamoto et al., 2000), *GFRα1*^*EGFP*^ (Uesaka et al., 2007) and *Nanos3*^*Cre*^ (Suzuki et al., 2008) alleles were described in the respective literature. The mice were heterozygous for one or two of these alleles and simply indicated by their allelic name(s), with the background of C57BL/6 (Japan CLEA and Japan SLC). Homozygous C57BL/6-Tg(UBC-GFP)30Scha/J (designated as *UBI-GFP*; Schaefer et al., 2001), C57BL/6-Tg (CAG-EGFP)C14-Y01-FM131Osb (designated as *CAG-EGFP*; Nakanishi et al., 2002) and WBB6F1-*Kit*^*W*/*Wv*^ mice were purchased from The Jackson Laboratory and Japan SLC, respectively. All animal experiments were conducted with approval of The Institutional Animal Care and Use Committee of National Institutes of Natural Sciences and The Hiroshima University Animal Research Committee.

### Method details

#### Immunofluorescence (IF)

IF of whole-mount seminiferous tubules stretched on slide glass was carried out as previous reported (Nakagawa et al., 2010) using anti-GFP Ab (Nacalai Tesque; 1:400), anti-GFRα1 Ab (R&D Systems; 1:400), anti-Rarγ Ab (Cell Signaling; 1:200), anti-Plzf Ab (Santa Cruz; 1:200), anti-KIT Ab (R&D systems; 1:500), and anti-SYCP3 Ab (Abcam; 1:500). Paraffin sections (7 μm-thick) of the testes were immunostained using anti-GFP Ab (Thermo Fisher; 1:300 or Abcam; 1:300), anti-Plzf Ab (EMD Millipore; 1:100), anti-SYCP3 Ab (Abcam; 1:500) and anti-Collagen IV (Abcam; 1:500) after antigen retrieval in 0.01 M citrate (pH 6.0; > 90C maintained for 10 min). All secondary antibodies were Alexa Fluor-conjugated, purchased from Thermo Fisher Scientific, and used at 1:400 dilutions. Slides were mounted in Fluoro-KEEPER Antifade Reagent (Nacalai Tesque). Observations and scoring were carried out using an Olympus BX51 upright fluorescence microscope equipped with a DP72 CCD camera, an Olympus FLUOVIEW FV3000 confocal laser scanning microscope, or a Leica TCS SP8 confocal system.

#### Pulse-transplantation experiments

*GFRα1*^*CreERT2*^; *CAG-CAT-EGFP* or *Ngn3-CreER*^*TM*^; *CAG-CAT-EGFP* mice were injected intraperitoneally with 2.0 mg of 4-hydroxytamoxifen (Sigma-Aldrich) per individual at the age of 3-4 months, as described before (Hara et al., 2014). After 2 days, donor mice were sacrificed and their testes were dispersed and processed for transplantation as described (Ogawa et al., 1997). Briefly, a single-cell suspension was prepared from their testes by enzymatic digestion using type IV collagenase, DNase I and type I-S hyaluronidase (Sigma-Aldrich). Singly dissociated testicular cells were injected into the seminiferous tubules of the host C57BL/6 mice which had been treated with busulfan intraperitoneally (44mg/kg; Wako) to deplete the endogenous germ cells completely, through the efferent ductulus 5 weeks prior to transplantation. For each host testis, 1 × 10^6^ unfractionated donor cells were injected, containing 1 × 10^3^ or 2 × 10^3^ GFP-labeled cells for GFRα1- or Ngn3-induction experiments, respectively. After 2-180 days, host testes were removed and the entire ~1.7 m-long tubules were recovered, stretched and fixed on a pair of slide glasses, and processed for whole-mount IF. The biological repeats are summarized in Table S2, and the raw counts are presented in Tables S1A–S1H.

#### Intravital live imaging

Testicular cell suspensions were prepared from *GFRα1*^*EGFP*^ pup mice (5-7 days post-partum), and injected into the tubules of 3-month-old *Kit*^*W/WV*^ mice (4 × 10^5^ donor cells per host testis). After 4 or 90 days, live-imaging of the testis of host mice that were kept under anesthesia was performed as described previously (Yoshida et al., 2007), using an Olympus IX61WI epifluorescence microscope. Time-lapse images were captured at the rate of one flame per 30 min using the Andor iXon EM-CCD camera controlled by Metamorph software (Molecular Devices). Movies were constructed by Metamorph software. An intercellular bridge was deemed to be intact if the cells remained within 30 mm for more than 6 h. The frequencies of cell division and syncytial fragmentation were measured from live-imaging data.

#### WIN18,446 treatment

Three-month-old busulfan-treated C57BL/6 mice were injected subcutaneously with 2 mg WIN18,446 (bisdichloroacetyldiamine; Cayman Chemical) dissolved in dimethyl sulfoxide (DMSO: 40 μg/μL; Nacalai Tesque) for 13 consecutive days from TP(–2) through to TP10, while transplantation was performed on TP0. The control DMSO injection did not contain WIN18,446. For fate analysis of GFRα1- or Ngn3-induced cells following transplantation, donor testis cell suspensions were prepared from 3-month-old *GFRα1*^*CreERT2*^; *CAG-CAT-EGFP* or *Ngn3-CreER*^*TM*^; *CAG-CAT-EGFP* mice, respectively, following administration of TM 2 days prior to dissociation and transplantation. 5 × 10^5^ cells were injected to each host testis. Two to sixty days after transplantation, host testes were collected and the entire ~1.7 m-long tubules were processed for whole-mount IF. At least 4 donor mice were used and 7 host testes were analyzed for each time point. For colony formation assay, testes cells of 3-month-old mice homozygous for *UBI-EGFP* were transplanted (5 × 10^5^ cells per recipient testis). After 60 days, host animals were sacrificed and their testes were removed for observation and photographing using a Leica M205C stereomicroscope equipped with a Leica DFC490 CCD camera under UV light. After macroscopic observation, the entire seminiferous tubules were processed for whole-mount IF to measure the GFP^+^ repopulating colonies. Some of the host animals were housed with C57BL/6 female mice in isolated cages for natural mating. The donor origin of the obtained pups was verified by fluorescence under UV light. At least 6 donor mice were used for transplantation and 15 host testes were analyzed.

### Biophysical model

#### 1. Model

##### Lattice representation of the seminiferous tubules

The model used here to describe the clonal dynamics of transplanted spermatogonial stem cells is based on a ‘voter-like’ model of stem cell dynamics originally developed for the homeostatic case (Hara et al., 2014; Klein et al., 2010): The seminiferous tubules are partitioned into a discrete lattice. Each lattice site can host up to one GFRα1^+^ syncytial unit; this is motivated by the largely constant stem cell density in the seminiferous tubules during homeostasis (Hara et al., 2014; see Figure 4A). GFRα1^+^ units are characterized by their syncytial state, i.e., A_s_, A_pr_, A_al–3_ etc. GFRα1^–^ cells, which arise through differentiation, are treated off-lattice, i.e., as a structureless pool of cells, since there is no apparent density constraint which would enforce similar restrictions on the number of units per lattice site. Hence, at each time point, the state of the system is given by the occupation of the lattice sites with GFRα1^+^ syncytia and the total number of GFRα1^–^ cells.

##### Cell fate dynamics

Cell fate processes like division and fragmentation are represented as Poisson processes, i.e., memoryless stochastic events with a fixed probability per unit time. The following four processes, summarized in Figure 4A, define the model dynamics:

• *Incomplete division.* Each GFRα1^+^ unit can undergo an incomplete division with transition rate λ.
• *Fragmentation and migration.* Each GFRα1^+^ unit of length *k* can undergo fragmentation with transition rate (*k* – 1)η, where η is the reference transition rate. Upon fragmentation, each syncytial bridge may break with probability 1/2. One random fragment remains at the original site whereas the other ones are randomly displaced to sites in the neighborhood. The neighborhood of a reference site is given by all lattice sites ± *r* sites in both directions. These events are coupled with the differentiation of the former occupant into a GFRα1^–^ unit (and therefore leaves the lattice), if an ‘invaded’ site already harbors a unit.
• *Death.* Each GFRα1^+^ unit can undergo cell death with a transition rate γ before the system reaches the time point $t=t_{0}$ (as measured from the time point of clonal transplantation), where $t_{0}$ is a parameter of the model. This process also effectively accounts for possible loss of a GFRα1^+^ unit through differentiation that is not compensated by the fragmentation of a neighboring GFRα1^+^ unit. After $t=t_{0}$, GFRα1^+^ units can only be lost through differentiation associated with the fragmentation and migration of neighbors.
• *Progenitor proliferation.* Each GFRα1^–^ cell proliferates at a transition rate μ. This process represents the net proliferation of the GFRα1^–^ compartment that results from cell division, fragmentation, cell death and possibly reversion to GFRα1^+^ cells (see Section ‘Possible role of reversion’ below for a discussion). The transition rate μ is an effective aggregate proliferation rate since we do not resolve the heterogeneity of the GFRα1^–^ compartment, which includes GFRα1^–^ A_undiff_ (largely Ngn3^+^), differentiating spermatogonia (undergoing serial mitotic divisions), spermatocytes (undergoing meiotic divisions) and haploid spermatids.
• *Possible role of reversion.* In experiments, we find a significant amount of GFRα1^+^ units even in the case of GFRα1^–^ transplants (Figure S3B), which suggests the presence of a certain degree of “reversion” from the GFRα1^–^ compartment to the GFRα1^+^ compartment, at least soon after transplantation. Albeit implicitly, our modeling scheme may effectively take into account such a reversion process: First, when predicting the clonal behavior of the GFRα1^–^ transplants, we used the experimental clonal distribution at day 2 to initialize the model (see Section ‘Simulation protocol’ above), so that any initial reversion up to day 2 would have already been taken into account. At later times, reversion would only alter the net flux between GFRα1^+^ and GFRα1^–^ compartments and would therefore be undetectable since its effect on clonal distributions could be captured by an altered gross, or “effective” rate of GFRα1^+^ cell differentiation.

#### 2. Comparison with experiments

##### Simulation protocol

To obtain dynamic clone size statistics, we compute a large number of stochastic realizations of the system using a standard stochastic simulation algorithm (Gillespie, 1977). To capture the post-transplantation dynamics of spermatogonial stem cells, we start simulations at time t=2 days after transplantation, with a small initial number of cells drawn from the experimentally determined clone size distribution at that time. Hence, all statistical quantities coincide with experimental data at day 2. From then on, we let the system evolve on its own according to the above dynamics.

##### Observables

Neglecting the spatial distribution of syncytia, the state of a clone at each time can be characterized by its distribution of GFRα1^+^ syncytia with different numbers of nuclei, $\vec{n}=\left(n_{1},n_{2},n_{3},\ldots \right)$, and the total number *m* of GFRα1^–^ cells, i.e., by the vector $\left(\vec{n},m\right)$. From a sufficiently large number of stochastic realizations of the system, the time-dependent size distribution of clones, $P\left(\vec{n},m,t\right)$, can be determined. To fit parameters and compare the resulting model predictions to experimental data, we analyzed the following statistical observables.

• *Survival rate of clones with GFRα1*^*+*^ *content.* The fraction of clones that contain at least one GFRα1^+^ cell (henceforth termed ‘persisting clones’) is given by

(S1) S(t)=1−P(0,t),

where P(n,t) is the marginal distribution of GFRα1^+^ cell number $n=\underset{k}{\sum }kn_{k}$, see Figure 4B and Figure S3A.

• *Persisting clone size distribution.* The subdistribution of GFRα1^+^ cells in the persisting population is given by

(S2) $$\tilde{P}\left(n,t\right){|}_{n\geq 1}=\frac{P\left(n,t\right)}{S\left(t\right)},$$

see Figure 4E and Figure S3D.

• *Average persisting clone size.* From the persisting clone size distribution Eq. (S2), we obtain the average persisting GFRα1^+^ cell content as

(S3) $$n\left(t\right)=\underset{n\geq 1}{\sum }n\tilde{P}\left(n,t\right),$$

see Figure 4C and Figure S3B.

• *Average total GFRα1*^*–*^ *progeny.* The average total GFRα1^–^ progeny generated by a clone (whether still containing GFRα1^+^ cells or not) is given by

(S4) $$\left\langle m\left(t\right)\right\rangle =\underset{\vec{n},m}{\sum }mP\left(\vec{n},m,t\right),$$

see Figure 4D and Figure S3C.

• *Average syncytial composition.* The average syncytial composition as a function of time is given by

(S5) $$\vec{n}\left(t\right)=\underset{\vec{n},m}{\sum }\vec{n}P\left(\vec{n},m,t\right),$$

from which the relative composition is obtained as

(S6) $$\vec{r}\left(t\right)=\frac{\left\langle \vec{n}\left(t\right)\right\rangle }{\sum_{k}\left\langle n_{k}\left(t\right)\right\rangle },$$

see Figure 4F and Figure S3E.

##### Parameter fits

A fit of the model parameters to experimental data is obtained as follows. The incomplete division rate λ is independently fixed by measurements from live imaging of GFRα1^+^ units (Figure 3). The remaining parameters (the fragmentation rate per intercellular bridge η, the effective loss rate during the initial loss phase, γ, and the length of the loss phase, $t_{0}$) are determined by a numerical fit of key observables as follows. To include information from both the relative composition and the absolute size of the persisting clones, as well as information about the total loss of clones with GFRα1^+^ content, we define the following residuals between simulation results and experimental data,

(S7) $$R_{1}=\underset{i}{\sum }{\left({\vec{r}}_{\text{sim}}\left(t_{i}\right)-{\vec{r}}_{\text{ex}}\left(t_{i}\right)\right)}^{2},$$

(S8) $$R_{2}=\underset{i}{\sum }{\left(1-\frac{n{\left(t_{i}\right)}_{\text{sim}}}{n{\left(t_{i}\right)}_{\text{ex}}}\right)}^{2},$$

(S9) $$R_{3}=\underset{i}{\sum }{\left(S_{\text{sim}}\left(t_{i}\right)-S_{\text{ex}}\left(t_{i}\right)\right)}^{2},$$

where the $t_{i}$ denote the measurement time points used for the fit (2, 6, 10, 14 and 20 days). We use these residuals to construct a single cost function that defines how well the simulation matches the experimental data,

(S10) $$C=\text{ln}\;R_{1}+\ln\;R_{2}+\ln\;R_{3}.$$

Regarding the cost *C* as a function of the parameter set, the best fit is defined as the parameter set that minimizes *C*.

Practically, this minimization is carried out using a Covariance Matrix Adaptation Evolution Strategy (CMA-ES) (Hansen et al., 2019). For each parameter set, 10^4^ realizations of the system are computed to obtain converged clone size distributions.

The list of fit parameters is provided in Table S4.

##### Sensitivity analysis

To obtain insight into how well the values of the fit parameters are constrained by the data, we compute the cost function *C*, defined in Eq. (S10), in a neighborhood around the best-fit values, varying all parameter values by ± 75% of the best-fit values. The results are shown in Figures S3F–S3H, displaying the cost function as density plots depending on pairwise combinations of the three parameters. Due to the definition of the cost function, an increase of *C* by ln(2) (as indicated by adjacent contours in Figures S3F–S3H) corresponds to the product of residuals given by Equations ((S7), (S8), (S9)) increasing by a factor of 2 and therefore to a substantial worsening of the fit. Thus, Figures S3F–S3H indicate that the loss rate during the initial loss phase, and length of the loss phase are well constrained within the given model paradigm, while the fragmentation rate shows some variability, with relative values varying by about ± 20% within the first contour (Figures S3F and S3H). Moreover, the plots reveal the interchangeability of parameters with respect to their effect on observed clonal properties: The fragmentation rate η has an effect largely independent of the initial loss phase-related parameters, as indicated by contours being nearly parallel to the parameter axes in Figures S3F and S3H. In contrast, the rate constant and length of the initial loss show a certain interchangeability as indicated by tilted contours in Figure S3G, as expected from their similar role in determining the overall magnitude of the initial loss.

##### Origin of minor discrepancies with the data

Although the model shows a highly accurate match with experimental data, there are some systematic discrepancies due to the simplified nature of the modeling scheme, including the following: First, we have implemented cell death in a simplified manner, imposing a constant rate of cell death that occurs over a fixed time period (fit to be around 8.1 days), followed by a complete absence of cell death. Therefore, after the initial period, the number of surviving clones will be predicted to remain constant within the framework of the model. However, experimentally, the number of surviving clones shows a slow decrease beyond TP8.1 until TP180 (Figures 2C and 4B; Figure S3A). It is likely that this slow decrease of surviving clone number reflects an infrequent but non-zero cell death contribution that continues beyond the initial 8.1 days. (For much longer times, the contribution of the merger of expanding clonal patches (colonies) cannot be ruled out.) Second, to simplify the modeling framework, we also suppose that the division rate of GFRα1^+^ cells is fixed over the time course up to TP30, and does not vary within the population. Moreover, we consider a purely stochastic pattern of cell division and differentiation, neglecting the observed spatiotemporal variations: In reality, the timing of GFRα1^+^ cell division, differentiation of GFRα1^+^ to GFRα1^–^ cells, as well as further differentiation steps show considerable levels of local synchronization within the tubules, in association with the “seminiferous epithelial cycle,” a periodic change of cell associations with 9-day cycle (reviewed in Yoshida, 2018). Together, these effects will have the effect of broadening the distribution of clone sizes, with some clones delayed in their expansion, while others may be accelerated. In other words, the simplified modeling scheme seeks to iron out these effects by imposing average rates for the processes of division, fragmentation and differentiation. Consistent with this, while the average clone sizes are accurately predicted over the time course (Figure 4C; Figure S3B), the model shows a somewhat narrower distribution than experiment, which becomes most pronounced at TP20 (Figure 4E; Figure S3D). It is notable that, at longer times (viz. TP30), where the effects of heterogeneities in division and differentiation would be expected to become suppressed by averaging the temporal fluctuations, the quality of the model predictions become enhanced. Overall, given the simplicity of the model and the complexity of the regeneration process following transplantation, we would argue that the agreement between the predictions of the model and the data is remarkably good.

### Quantification and statistical analysis

#### Scoring the GFP^+^ clonal clusters

The entire seminiferous tubules that comprise the individual host testes were triple-stained for GFP/GFRα1/Plzf or GFP/GFRα1/Rarγ by whole-mount IF method. After all clonal clusters of GFP^+^ cells in individual host testes were identified visually using Olympus BX51 microscopy, the detailed compositions of each clones were scored using a Leica TCS SP8 confocal system.

For samples from TP2 to TP20, the numbers and unit composition (A_s_, A_pr_ or A_al_) of GFRα1^+^ A_undiff_, GFRα1^–^ A_undiff_, and more advanced differentiating cells (differentiating spermatogonia, spermatocytes and spermatids) were scored (Tables S1A and S1G). In some samples, the numbers of GFRα1^+^ A_undiff_, GFRα1^–^ A_undiff_ and differentiating cells (Table S1B; without considering their unit composition), or the numbers and compositions of GFRα1^+^ A_undiff_ and GFRα1^–^ cells (Table S1C; without classifying into A_undiff_ and differentiating cells), or the numbers of GFRα1^+^ A_undiff_ and GFRα1^–^ cells (Table S1D). For samples from TP30 and longer times after transplantation, in which the observed clones were too large to count all the constituent GFP^+^ cells, the lengths of the repopulating colonies (physical extension of clones) were measured using a CellSens Standard software (Olympus) on images acquired using an BX51 fluorescence microscope equipped with a DP72 CCD camera (Olympus), in addition to scoring the unit compositions (Table S1E), total number (Table S1F), or presence/absence (Table S1H) of GFRα1^+^ A_undiff_ including in individual clones. For Tables S1A and S1B, GFRα1^+^/Plzf^+^ cells, GFRα1^–^/Plzf^+^ cells and GFRα1^–^/Plzf^–^ cells were defined as GFRα1^+^ A_undiff_, GFRα1^–^ A_undiff_, and more advanced cells, respectively; no GFRα1^+^/Plzf^–^ cells were observed. For Table S1G (WIN-treated experiments), GFRα1^–^/Rarγ^+^ and GFRα1^–^/Rarγ^–^ cells were defined as GFRα1^–^ A_undiff_ and differentiating cells, respectively. After these observations, some specimens were subsequently stained for c-Kit or SYCP3, to determine the differentiation status of GFP^+^ clones consisting only of differentiating cells.

These datasets were used in each analysis, so that maximum numbers of data points were included as summarized in Table S2.

#### Analysis of the A_undiff_ composition in long-term repopulating colonies

The testicular cells (5 × 10^5^ cells) of 3-month-old mice homozygous for *CAG-EGFP* were transplanted to each testis of busulfan-treated hosts. After 180 days, the host seminiferous tubules were triple-stained for GFP, GFRα1 and Rarγ by whole-mount IF method. The lengths of the GFP^+^ repopulating colonies were measured by the same procedure described above. The numbers of GFRα1^+^/Rarγ^–^, GFRα1^+^/Rarγ^+^ and GFRα1^–^/Rarγ^+^ cells included in individual colonies were scored using an Olympus FLUOVIEW FV3000 confocal laser scanning microscope. The results are summarized in Table S3.

#### Labeling efficiencies

For GFRα1-induction experiments, the labeling efficiencies of *GFRα1*^*CreERT2*^; *CAG-CAT-EGFP* mice was calculated 2 days after administration of 4OH-tamoxifen. The frequencies of GFRα1-positive spermatogonia in labeled cells were determined using whole-mount double IF for GFRα1 (R&D Systems; 1:400) and GFP (Nacalai Tesque; 1:400) on seminiferous tubules from the induced *GFRα1*^*CreERT2*^; *CAG-CAT-EGFP* mice. The labeling efficiency in GFRα1-induction experiments was calculated as 27.8% (1,529 / 5,499). For Ngn3-induction experiments, the labeling efficiencies after pulse-labeling of *Ngn3-CreER*^*TM*^; *CAG-CAT-EGFP* was calculated for A_s_ fraction, based on the frequencies of the labeled cells and the *Ngn3*-EGFP-positive spermatogonia as described (Nakagawa et al., 2007). These frequencies were determined as relative occurrences compared to E-cadherin^+^ A_s_ using whole-mount double IF for E-cadherin (Takara Bio; 1:1,000) and GFP (Nacalai Tesque; 1:400) on seminiferous tubules from *Ngn3-EGFP* mice and the induced *Ngn3-CreER*^*TM*^; *CAG-CAT-EGFP* mice. The frequencies of the labeled A_s_ and the *Ngn3*-EGFP-positive A_s_ were 18.7% (942 / 14,154) and 6.24% (1,875 / 8,135), respectively. Hence, the labeling efficiency in Ngn3-induction experiments was estimated as 33.3%.

#### The number of labeled cells injected

Total number of GFRα1^+^ spermatogonia per testis was calculated to be 57,800 cells, based on the density of GFRα1^+^ cells (34 cells/mm; Hara et al., 2014) and the entire length of seminiferous tubules measured in this study (about 1,700 mm; Nakagawa et al., 2007). On the other hand, total number of Ngn3^+^ spermatogonia per testis was estimated to be 73,135 cells by the percentages of Rarγ^+^ A_undiff_ and GFRα1^+^ A_undiff_ in total A_undiff_ (62% and 49%, respectively; Ikami et al., 2015), given that Ngn3^+^ spermatogonia exclusively express Rarγ (Ikami et al., 2015). Individual testes of *GFRα1*^*CreERT2*^; *CAG-CAT-EGFP* and *Ngn3-CreER*^*TM*^; *CAG-CAT-EGFP* mice contained approximately 18.4 × 10^6^ cells. In this study, 1 × 10^6^ testicular cells with 98.5% viability was injected each host testis. Therefore, the numbers of GFRα1^+^ and Ngn3^+^ spermatogonia injected per testis were calculated to be 3,094 and 3,915 cells, respectively. The number of labeled cells in GFRα1-induction experiments was calculated as 1,070 cells, based on its labeling efficiency (27.8%) and the percentage of GFRα1^+^ cells in labeled cells (80.4%). For Ngn3-induction experiments, the calculated number of labeled cells injected per host testis was 1,977 cells on the basis of its labeling efficiency (33.3%) and the percentage of Rarγ^+^ A_undiff_ in labeled cells (66.0%).

#### Evaluation of the potential merger of repopulating colonies

Our analysis of the clonal dynamics relies on the integrity of clonal assignment. Therefore, during transplantation, since spermatogonia settle randomly onto the seminiferous tubules, it’s important to consider the potential for chance merger events, where non-clonally related GFP^+^ cells and syncytia spatially overlap. An estimate for the probability of clone merger can be made based on the following argument: Let us suppose that, following transplantation, there are an average of N individual GFP^+^ cell clusters, positioned randomly along a length, L, of seminiferous tubule, each of which has a typical (i.e., average) spatial extent, a, along the tubule length. In this case, the probability that a given cluster is merged with another is given approximately as $p=1-{\left(1-a/L\right)}^{N-1}$. With the clonal data obtained from tubules of length L=1,700mm, we then estimate p=0.004 (viz. 0.4%) on TP30 (when N=6 and a=1.2mm), p=0.01 (viz. 1%) on TP90 (when N=3 and a=6mm), and p=0.01 (viz. 1%) on TP180 (when N=4 and a=8.4mm). Therefore, although the actual merger probabilities may be somewhat larger, given that donor cell suspension cannot be injected evenly over the entire seminiferous tubules, such potential merger events are sufficiently rare that they would have only a minimal impact on the clone data analyses.

#### Statistical analysis

Sample numbers and experimental repeats are indicated in figure legends and Table S2. All data are presented as mean ± SEM. For comparing two datasets, an unpaired t test was used. Significance summary: p > 0.05 (ns), ^∗^p ≤ 0.05, ^∗∗^p ≤ 0.01, and ^∗∗∗^p ≤ 0.001.

## Supplemental information

Document 1. Figures S1–S4 and Tables S3–S5

Table S1. The complete set of clonal data of GFRα1- and Ngn3-induced donor spermatogonia following transplantation, related to Figures 1-6, Figures S2-S4, and Table S2 Related to Figures 1, 2, 3, 4, 5, 6, and S2–S4 and Table S2 (A) Clonal data of GFRα1- and Ngn3-induced donor spermatogonia on TP2-20, in the number and that composition of syncytial units of GFRα1+ Aundiff, GFRα1– Aundiff, and differentiating cells (differentiating spermatogonia, spermatocytes, and spermatids), related to Figures 1, 2, 3, 4, S2, and S3 and Table S2. (B) Clonal data of GFRα1- and Ngn3-induced donor spermatogonia, respectively, in the number of GFRα1+ Aundiff, GFRα1– Aundiff, and differentiating cells, related to Figures 2 and S2 and Table S2. (C) Clonal data of GFRα1-induced donor spermatogonia, in the composition of syncytial units of GFRα1+ Aundiff and GFRα1– cells, related to Figures 2, 3, 4, and S2 and Table S2. (D) Clonal data of GFRα1- and Ngn3-induced donor spermatogonia, in cell number of GFRα1+ Aundiff and GFRα1– cells, related to Figures 2 and S2 and Table S2. (E) Clonal data of GFRα1- and Ngn3-induced donor spermatogonia on TP30 to TP180, in the composition of syncytial units of GFRα1+ Aundiff and spatial extent in length, related to Figures 1, 2, 3, 4, S2, and S3 and Table S2. (F) Clonal data of GFRα1- and Ngn3-induced donor spermatogonia on TP30 to TP90, in cell number of GFRα1+ Aundiff and spatial extent in length, related to Figures 2, S2, and S3 and Table S2. (G) Clonal data of GFRα1- and Ngn3-induced donor spermatogonia following transplantation into donors treated with DMSO (control) or WIN, on TP2 to TP20, in the composition of syncytial units of GFRα1+ /Rarγ– Aundiff, GFRα1+ /Rarγ+ Aundiff, GFRα1– /Rarγ+ Aundiff, GFRα1– /Rarγ– cells, related to Figures 5 and S4 and Table S2. (H) Clonal data of GFRα1- and Ngn3-induced donor spermatogonia following transplantation into DMSO (control) or WIN treated hosts on TP60, in spatial extent in length and the presence or absence of GFRα1+ cells, related to Figure 5 and Table S2.

Table S2. Summary of the data set used for individual data analyses, and biological repeats therein, related to Figures 1-6, Figures S2-S4, and Tables S1A-S1H

Document S2. Article plus supplemental information
