# Supplementary material for: Transient suppression of transplanted spermatogonial stem cell differentiation restores fertility in mice
Source: Cell Stem Cell. 2021 Aug 5;28(8):1443–1456.e7. doi: 10.1016/j.stem.2021.03.016 (PMC8351876; doi:10.1016/j.stem.2021.03.016)
Supplement: Document 1. Figures S1–S4 and Tables S3–S5 [file mmc1.pdf]

Cell Stem Cell, Volume *28*

Supplemental Information

Transient suppression of transplanted spermatogonial stem cell differentiation restores fertility in mice

Yoshiaki Nakamura, David J. Jörg, Yayoi Kon, Benjamin D. Simons, and Shosei Yoshida

Cell Stem Cell, Volume *28*

Supplemental Information

Transient suppression of transplanted spermatogonial stem cell differentiation restores fertility in mice

Yoshiaki Nakamura, David J. Jörg, Yayoi Kon, Benjamin D. Simons, and Shosei Yoshida

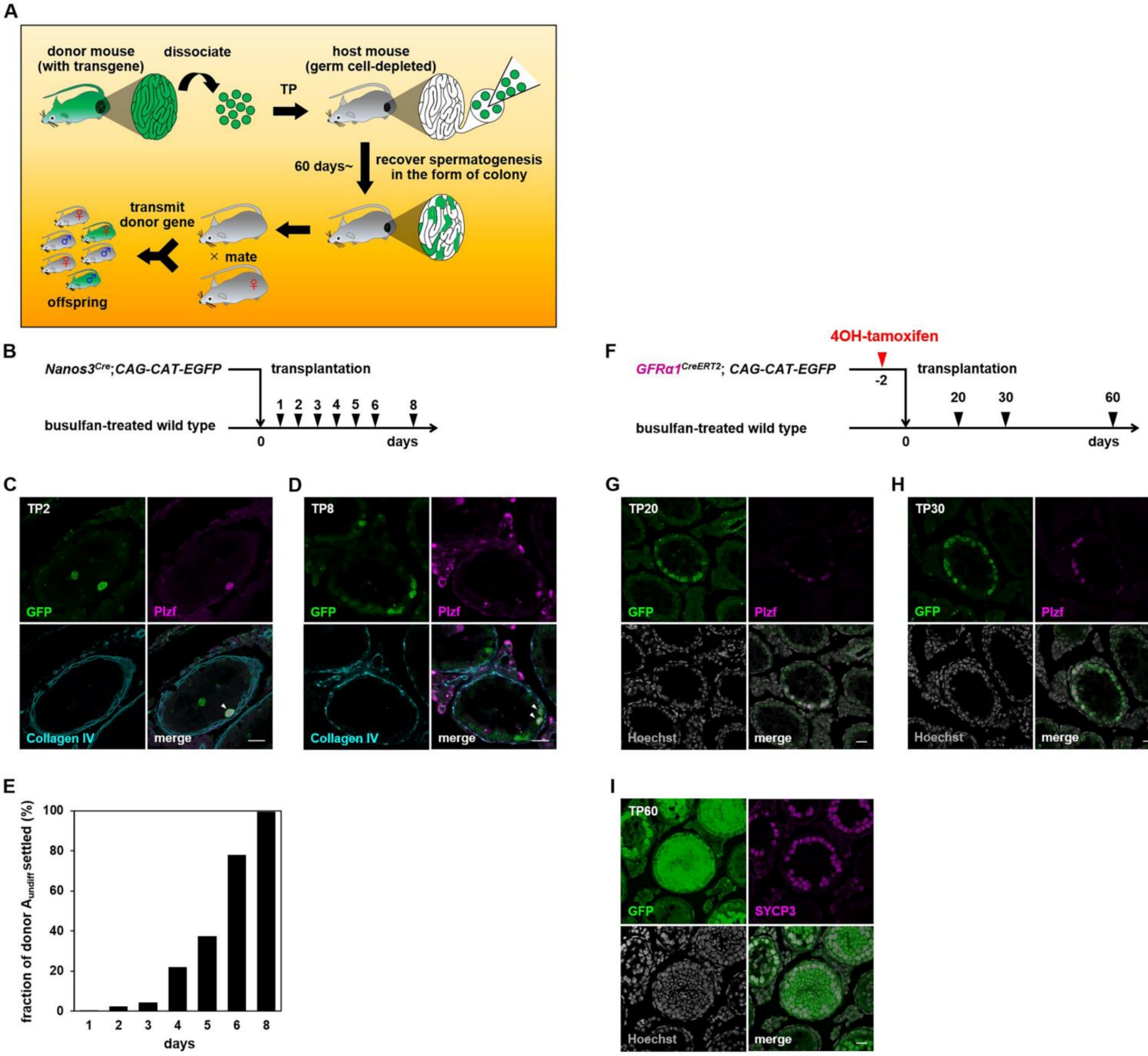
A
donor mouse (with transgene)
dissociate
host mouse (germ cell-depleted)
TP
60 days~
recover spermatogenesis in the form of colony
transmit donor gene
× mate
offspring
B
Nanos3Cre;CAG-CAT-EGFP
transplantation
busulfan-treated wild type
0
1 2 3 4 5 6 8
days
C
TP2
GFP
Plzf
Collagen IV
merge
D
TP8
GFP
Plzf
Collagen IV
merge
E
fraction of donor Aundiff settled (%)
100
80
60
40
20
0
1 2 3 4 5 6 8
days
F
4OH-tamoxifen
GFRα1CreERT2; CAG-CAT-EGFP
-2
transplantation
20 30 60
busulfan-treated wild type
0
days
G
TP20
GFP
Plzf
Hoechst
merge
H
TP30
GFP
Plzf
Hoechst
merge
I
TP60
GFP
SYCP3
Hoechst
merge

Figure S1. Spermatogonial transplantation and repopulation process of donor A_{undiff} in host seminiferous tubules, Related to Figure 1.

(A) Outline of spermatogonial transplantation method in mouse. A single-cell suspension is prepared from donor testes and microinjected into the lumen of seminiferous tubules of germ cell-depleted host mice. Then, cells with SSC activity produce colonies that repopulate spermatogenesis. When donor testis cells carry genetic markers such as GFP, colonies of donor-derived spermatogenesis are identified easily in host testis by GFP florescence. Mating the host males to wild type females would produce marker-positive offspring, if enough number of sperm are produced in host testes based on prominent reconstitution of spermatogenesis. If the degree of repopulation is too low to restore the host fertility, however, intracytoplasmic sperm injection (ICSI) or round spermatid injection (ROSI) needs to be applied to generate pups, which requires sacrificing the host animals or remove their testes (Modified from Brinster, 2002, with permission). **(B)** Experimental schedule to analyze the translocation of donor A_{undiff} onto the basement membrane following transplantation (C and D). Note that all germ cells of *Nanos3Cre*; *CAG-CAT-EGFP* donor mice are labeled with GFP expression. **(C and D)** IF images of cross-section of host testis stained for GFP, Plzf and Collagen IV (visualizing basement membrane) on TP2 (C) and TP8 (D). Arrowheads indicate examples of $GFP^{+}/Plzf^{+}$ donor A_{undiff}. **(E)** Percentage of cells attached to the basement membrane out of the total $GFP^{+}/Plzf^{+}$ donor A_{undiff} at the indicated time points. **(F)** Experimental schedule for G-I. **(G-I)** IF images of a cross-section of host testes on TP20 (G), TP30 (H) and TP60 (I), stained for GFP (green), Plzf or SYCP3 (magenta) and DNA (using Hoechst 33442; grey scale). Scale bars indicate 25 µm.

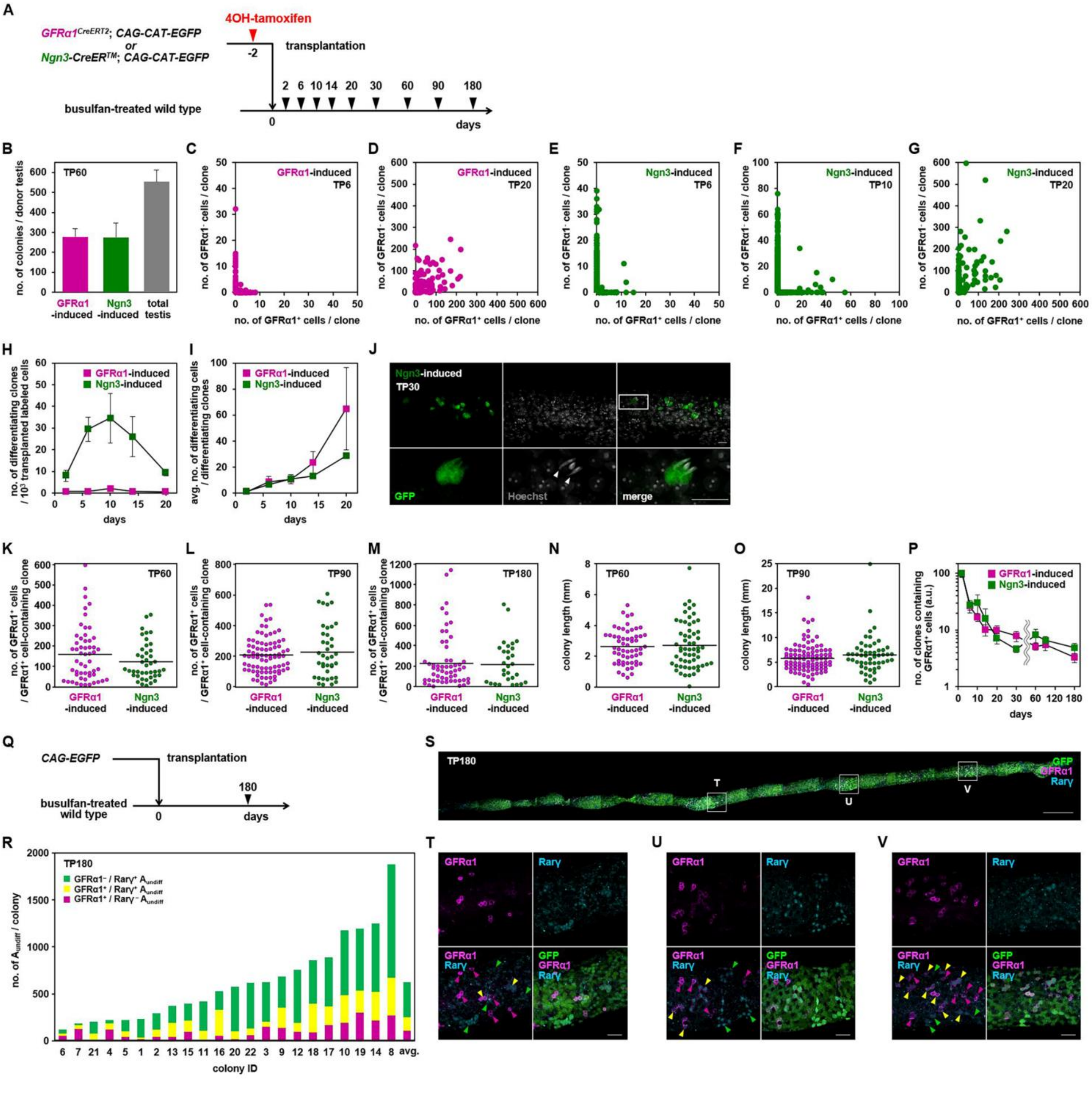

A
4OH-tamoxifen
GFRα1CreERT2; CAG-CAT-EGFP
or
Ngn3-CreERTM; CAG-CAT-EGFP
-2
transplantation
busulfan-treated wild type
0
2 6 10 14 20 30 60 90 180
days
B
TP60
no. of colonies / donor testis
GFRα1 -induced
Ngn3 -induced
total testis
C
GFRα1-induced TP6
D
GFRα1-induced TP20
E
Ngn3-induced TP6
F
Ngn3-induced TP10
G
Ngn3-induced TP20
no. of GFRα1⁻ cells / clone
no. of GFRα1⁺ cells / clone
H
GFRα1-induced
Ngn3-induced
no. of differentiating clones / 10³ transplanted labeled cells
days
I
avg. no. of differentiating cells / differentiating clones
days
J
Ngn3-induced
TP30
GFP
Hoechst
merge
K
TP60
no. of GFRα1⁺ cells / GFRα1⁺ cell-containing clone
L
TP90
M
TP180
N
TP60
colony length (mm)
O
TP90
colony length (mm)
P
no. of clones containing GFRα1⁺ cells (a.u.)
days
Q
CAG-EGFP
transplantation
busulfan-treated wild type
0
180
days
R
TP180
GFRα1⁻ / Rarγ⁺ Aundiff
GFRα1⁺ / Rarγ⁺ Aundiff
GFRα1⁺ / Rarγ⁻ Aundiff
no. of Aundiff / colony
6 7 21 4 5 1 2 13 15 11 16 20 22 3 9 12 18 17 10 19 14 8 avg.
colony ID
S
TP180
GFP
GFRα1
Rarγ
T
U
V

Figure S2. Supplemental data for post-transplantation dynamics of GFRα1- and Ngn3-induced cells, Related to Figure 2.

(A) Experimental schedule for B-P, to study the dynamics of transplanted GFRα1- and Ngn3-induced cells. **(B)** Numbers of GFRα1- or Ngn3-induced cell-derived repopulating colonies normalized per donor testis, compared with those derived from total testicular cells, 60 days after transplantation. **(C-G)** Distribution of the numbers of GFRα1^{+}- and GFRα1^{-} cells in each clone until 20 days after GFRα1- (C and D) and Ngn3- (E-G) induced cells were transplanted. **(H)** Numbers of clones containing only differentiating cells (differentiating spermatogonia and more advanced cells) derived from GFRα1- and Ngn3-induced cells, normalized for 10^3 transplanted labeled cells. **(I)** Average numbers of differentiating cells (described above) per such differentiating clone. **(J)** Representative images of a Ngn3-induced clone (GFP^{+}) on TP30, showing synchronous differentiation to spermatids. A part of fragmented clones in upper right panel (square) is magnified in bottom panels. Note the condensed and elongated nucleus visualized by Hoechst 33342, characteristic for elongating spermatids. **(K-M)** The numbers of GFRα1^{+} cells included in each clone originated from GFRα1- and Ngn3-induced cells on TP60 (K), TP90 (L) and TP180 (M). **(N and O)** The length distribution of GFRα1- and Ngn3-induced colonies on TP60 (N) and TP90 (O). **(P)** Kinetics of the average number of GFRα1- and Ngn3-induced clones containing GFRα1^{+} cells, shown by arbitrary unit (a.u.) relative to the TP2 value (set to 100). **(Q)** Experimental schedule for (R-V), analyzing the composition of A_{undiff} in individual repopulating colonies. **(R)** Numbers of GFRα1^{+}/Rarγ^{-} (magenta), GFRα1^{+}/Rarγ^{+} (yellow), and GFRα1^{-}/Rarγ^{+} (green) A_{undiff} included in each of 22 colonies analyzed on TP180. The averaged values are also shown (avg.) and the raw data are in Table S3. It is highly likely that these include significant numbers of both GFRα1- and Ngn3-induced colonies: For example, if we assume, based on (B), that 50 % each of the colonies are derived from GFRα1- and Ngn3-induced founder cells, respectively, the possibility that 5 or more colonies originated from GFRα1- and Ngn3-cells have been analyzed will be calculated as $1 - 2(\sum_{n=0}^{4} {}_{22}C_n)/2^{22} = 0.996$. **(S)** A part of a seminiferous tubule at TP180, showing the entirety of a GFP^{+} donor cell-derived colony triple-stained for GFP, GFRα1, and Rarγ. **(T-V)** Magnified panels in regions indicated in (S). Arrowheads with magenta, yellow, and green indicate GFRα1^{+}/Rarγ^{-}, GFRα1^{+}/Rarγ^{+}, and GFRα1^{-}/Rarγ^{+} A_{undiff}, respectively. Values in B, H, I and P are shown in averages ± SEM; bars in K-O indicate the average. Scale bars, 25 µm (J), 500 µm (S), and 50 µm (T-V).

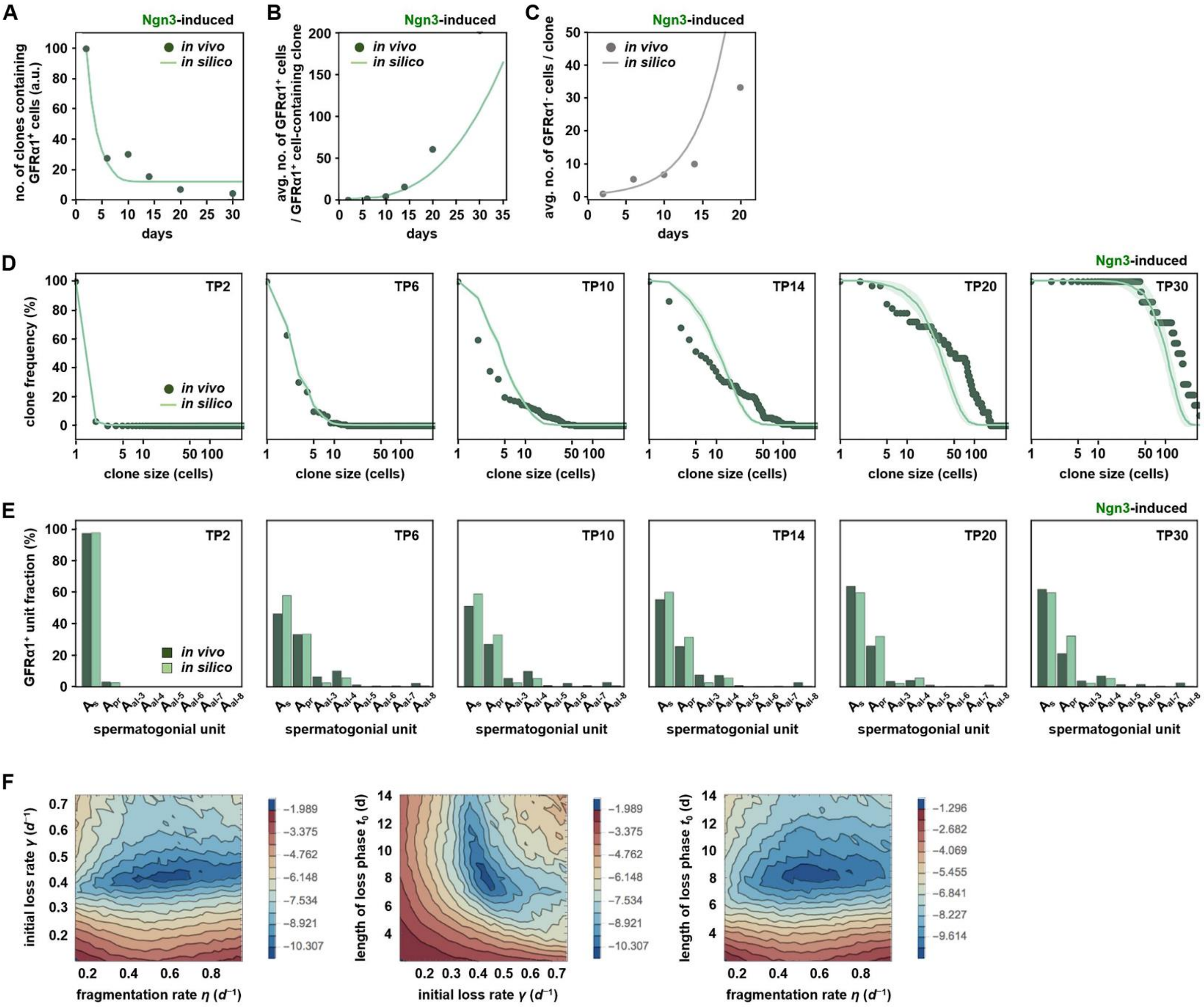

A
Ngn3-induced
in vivo
in silico
no. of clones containing GFRα1+ cells (a.u.)
days
B
Ngn3-induced
avg. no. of GFRα1+ cells / GFRα1+ cell-containing clone
C
Ngn3-induced
avg. no. of GFRα1- cells / clone
D
Ngn3-induced
TP2
TP6
TP10
TP14
TP20
TP30
clone frequency (%)
clone size (cells)
E
Ngn3-induced
GFRα1+ unit fraction (%)
spermatogonial unit
F
initial loss rate γ (d−1)
fragmentation rate η (d−1)
length of loss phase t0 (d)
initial loss rate γ (d−1)
length of loss phase t0 (d)
fragmentation rate η (d−1)

Figure S3. Supplemental data for model prediction of post-transplantation fate behavior of Ngn3-induced donor cells, Related to Figure 4 and STAR Methods.

(A and B) Number of clones containing at least one GFRα1$^+$ cells relative to TP2 (A), and the average number of GFRα1$^+$ cells contained in these clones (B), after Ngn3-induced cells were transplanted (dots: experiments, curve: model). **(C)** Average number of GFRα1$^-$ cells per clone after Ngn3-induced cells were transplanted (dots: experiments, curve: model). **(D)** Cumulative clone size distribution of GFRα1$^+$ cells among persisting clones after Ngn3-nduced cells were transplanted (dots: experiments, curve: model). **(E)** Composition of GFRα1$^+$ syncytia over time averaged over persisting clones after Ngn3-induced cells were transplanted (dark: experiment shown in Figure 3G, bright: model). **(F-H)** Parameter sensitivity analysis. Density plots show the dependence of the cost function used to perform parameter fits, given by Eq. (S10 in STAR Methods), on pairwise combinations of the three fit parameters. The best-fit values are located at the center of each plot; parameter axes span ± 75% of the best-fit values. Between adjacent contours, the cost function increases by $\ln(2) \simeq 0.693$ as indicated by the legends.

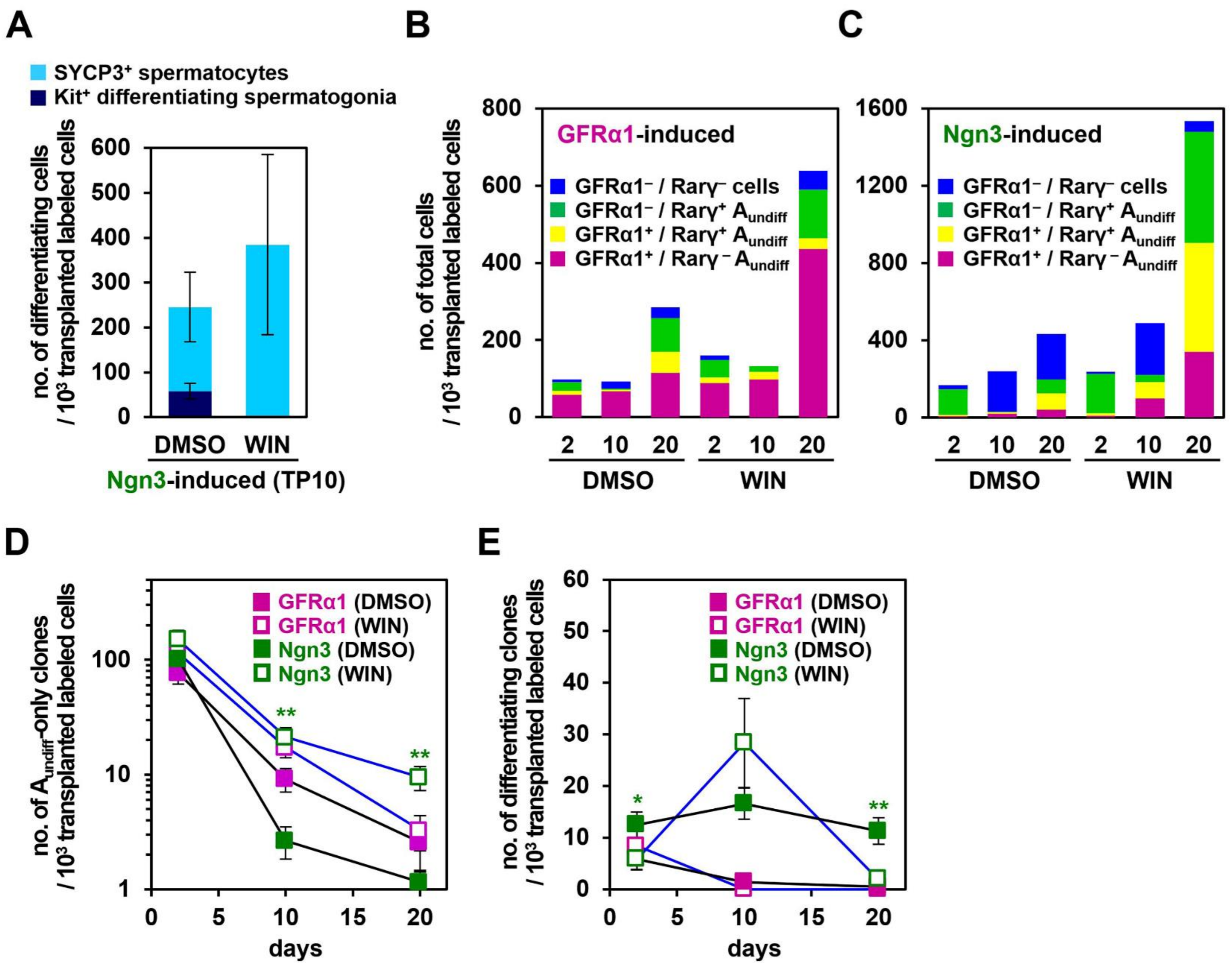
A
SYCP3+ spermatocytes
Kit+ differentiating spermatogonia
no. of differentiating cells / 10³ transplanted labeled cells
600
500
400
300
200
100
0
DMSO
WIN
Ngn3-induced (TP10)
B
GFRα1-induced
no. of total cells / 10³ transplanted labeled cells
800
600
400
200
0
GFRα1− / Rarγ− cells
GFRα1− / Rarγ+ Aundiff
GFRα1+ / Rarγ+ Aundiff
GFRα1+ / Rarγ− Aundiff
2
10
20
DMSO
WIN
C
Ngn3-induced
1600
1200
800
400
0
GFRα1− / Rarγ− cells
GFRα1− / Rarγ+ Aundiff
GFRα1+ / Rarγ+ Aundiff
GFRα1+ / Rarγ− Aundiff
2
10
20
DMSO
WIN
D
no. of Aundiff-only clones / 10³ transplanted labeled cells
100
10
1
GFRα1 (DMSO)
GFRα1 (WIN)
Ngn3 (DMSO)
Ngn3 (WIN)
**
**
0
5
10
15
20
days
E
no. of differentiating clones / 10³ transplanted labeled cells
60
50
40
30
20
10
0
GFRα1 (DMSO)
GFRα1 (WIN)
Ngn3 (DMSO)
Ngn3 (WIN)
*
**
0
5
10
15
20
days

Figure S4. Effect of temporary block of RA synthesis with WIN18,446 on donor cells, Related to Figure 5

(A) Compositions of differentiating cells originated from Ngn3-induced donor cells observed at TP10, with DMSO (control) or WIN treatment, classified into Kit^+ differentiating spermatogonia (cyan) and $SYCP3^+$ spermatocytes (indigo), normalized for 10^3 labeled cells transplanted. Total of 970 and 1521 differentiating cells from 4 donor testes were analyzed for DMSO and WIN-treated conditions, respectively, and shown as average ± SEM. **(B and C)** Numbers of GFRα1- (B) and Ngn3- (C) induced cells, observed in the host seminiferous tubules, with DMSO (controls) or WIN treatment at indicated time points, classified into $GFR\alpha1^+/Rar\gamma^-$ A_{undiff} (magenta), $GFR\alpha1^+/Rar\gamma^+$ A_{undiff} (yellow), $GFR\alpha1^-/Rar\gamma^+$ A_{undiff} (green), and $GFR\alpha1^-/Rar\gamma^-$ differentiating cells (blue; including differentiating spermatogonia, spermatocytes, and spermatids). **(D and E)** Numbers of clones composed only of A_{undiff} (D) or differentiating cells (i.e., Kit^+ differentiating spermatogonia and more advanced cells) (E), derived from GFRα1- or Ngn3-induced cells, with DMSO (controls) or WIN treatment, normalized for 10^3 transplanted labeled cells. Values are shown in averages ± SEM. $^{*}p < 0.05$, $^{**}p < 0.01$, or $^{***}p < 0.001$ between DMSO and WIN treatments.

Table S3. Numbers of GFRα1^{+} /Rarγ^{-} A_{undiff}, GFRα1^{+} /Rarγ^{+} A_{undiff}, and GFRα1^{-} /Rarγ^{+} A_{undiff} in individual repopulating clones derived from *CAG-EGFP* donor testes, analyzed on TP180, related to Figures S2Q-S2V and STAR Methods.

clone ID	numbers of cells				clone length (μm)
	GFRα1^{+} /Rarγ^{-} A_{undiff}	GFRα1^{+} /Rarγ^{+} A_{undiff}	GFRα1^{-} /Rarγ^{+} A_{undiff}	total	
1	15	18	198	231	3085.01
2	37	80	172	289	5391.1
3	148	54	420	622	8878.59
4	119	65	32	216	12220.72
5	41	60	116	217	3200.4
6	53	23	41	117	5152.78
7	124	39	20	183	5481.42
8	265	407	1207	1879	12072.13
9	135	218	329	682	5935.73
10	189	292	693	1174	13435.44
11	10	96	309	415	6748.89
12	92	96	565	753	11744.43
13	41	149	176	366	10858.55
14	214	304	727	1245	10429.12
15	94	117	181	392	8241.41
16	49	280	195	524	10524.01
17	166	199	519	884	7061.06
18	86	307	464	857	6666.57
19	296	234	662	1192	12282.1
20	15	86	470	571	7820.94
21	9	67	126	202	10009.36
22	55	72	488	615	8499.17

Table S4. Parameters used for the model compared with those for homeostasis, related to STAR Methods, Figure 4 and Figure S3.

parameters	description	value	obtained from	value	obtained from
		post-transplantation (this study)		homeostasis (Hara et al. 2014)	
**GFRα1$^+$ compartment**					
$\boldsymbol{\lambda}$	incomplete division rate	1/(3 days)	live imaging	1/(10 days)	live imaging
$\boldsymbol{\eta}$	fragmentation rate per intercellular bridge	1/(1.9 days)	fit	1/(20 days)	live imaging
$\boldsymbol{\gamma}$	effective loss rate during initial loss phase	1/(2.4 days)	fit	–	–
$\boldsymbol{t_0}$	length of initial loss phase	8.1 days	fit	–	–
**r**	maximum migration range upon fragmentation	2 sites	estimate	*2 sites	estimate
**GFRα1$^-$ compartment**					
$\boldsymbol{\mu}$	effective proliferation rate	1/(4.2 days)	fit	**not defined	–
**model geometry**					
$\boldsymbol{R}$	circumference of the tubules	5 sites	Klein et al. (2010)	5 sites	Klein et al. (2010)

*Biased migration along the tubule length is introduced based on the clone morphology and vasculature arrangement although the model dynamics depends only weakly on this parameter.

** GFRα1$^-$ cells do indeed undergo incomplete division. However, given that syncytial fragmentation within this population occurs very infrequently, their proliferation does not impact on the number of syncytial units; although it does of course increase the constituent cell number. Since Hara et al. (2014) sought to define the homeostatic dynamics, this study only considered the GFRα1$^-$ syncytial unit (rather than cell) number. Moreover, this study did not take into account the reverse transitioning from GFRα1$^-$ to GFRα1$^+$ states since such processes are similarly infrequent. Therefore, the rate of incomplete division was not defined.

Table S5. Evaluation of fertility of the host mice treated with DMSO (control) or WIN, related to Figure 6

treatment	ID	first offspring (term)	avg. litter size	no. of offspring	no. of GFP^{+} offspring	donor-derived offspring (%)
DMSO	DMSO-1	–	–	–	–	–
	DMSO-2	–	–	–	–	–
	DMSO-3	–	–	–	–	–
	DMSO-4	–	–	–	–	–
	DMSO-5	–	–	–	–	–
	DMSO-6	–	–	–	–	–
	DMSO-7	–	–	–	–	–
	DMSO-8	–	–	–	–	–
	DMSO-9	–	–	–	–	–
	DMSO-10	–	–	–	–	–
WIN	WIN-1	–	–	–	–	–
	WIN-2	214 days	8.3	33	33	100
	WIN-3	259 days	7.5	45	45	100
	WIN-4	–	–	–	–	–
	WIN-5	–	–	–	–	–
	WIN-6	–	–	–	–	–
	WIN-7	195 days	7.6	61	61	100
	WIN-8	–	–	–	–	–
	WIN-9	–	–	–	–	–
	WIN-10	–	–	–	–	–
	WIN-11	330 days	5.7	34	34	100
	WIN-12	331 days	7.1	50	50	100

Shown are the results of three separate experiments.
